# Cystatin A, a Potential Common Link for Mutant Myocilin Causative Glaucoma

**DOI:** 10.1371/journal.pone.0036301

**Published:** 2012-05-15

**Authors:** K. David Kennedy, S. A. AnithaChristy, LaKisha K. Buie, Teresa Borrás

**Affiliations:** Department of Ophthalmology, University of North Carolina School of Medicine, Chapel Hill, North Carolina, United States of America; Institut Jacques Monod, France

## Abstract

Myocilin (MYOC) is a 504 aa secreted glycoprotein induced by stress factors in the trabecular meshwork tissue of the eye, where it was discovered. Mutations in *MYOC* are linked to glaucoma. The glaucoma phenotype of each of the different *MYOC* mutation varies, but all of them cause elevated intraocular pressure (IOP). In cells, forty percent of wild-type MYOC is cleaved by calpain II, a cysteine protease. This proteolytic process is inhibited by MYOC mutants. In this study, we investigated the molecular mechanisms by which MYOC mutants cause glaucoma. We constructed adenoviral vectors with variants Q368X, R342K, D380N, K423E, and overexpressed them in human trabecular meshwork cells. We analyzed expression profiles with Affymetrix U133Plus2 GeneChips using wild-type and null viruses as controls. Analysis of trabecular meshwork relevant mechanisms showed that the unfolded protein response (UPR) was the most affected. Search for individual candidate genes revealed that genes that have been historically connected to trabecular meshwork physiology and pathology were altered by the *MYOC* mutants. Some of those had known *MYOC* associations (*MMP1, PDIA4, CALR, SFPR1)* while others did not (*EDN1, MGP, IGF1, TAC1*). Some, were top-changed in only one mutant (*LOXL1, CYP1B1, FBN1*), others followed a mutant group pattern. Some of the genes were new (*RAB39B, STC1, CXCL12, CSTA*). In particular, one selected gene, the cysteine protease inhibitor cystatin A (*CSTA*), was commonly induced by all mutants and not by the wild-type. Subsequent functional analysis of the selected gene showed that CSTA was able to reduce wild-type MYOC cleavage in primary trabecular meshwork cells while an inactive mutated CSTA was not. These findings provide a new molecular understanding of the mechanisms of MYOC-causative glaucoma and reveal CSTA, a serum biomarker for cancer, as a potential biomarker and drug for the treatment of MYOC-induced glaucoma.

## Introduction

The secreted glycoprotein, myocilin (MYOC), was identified in human trabecular meshwork (HTM) cells after prolonged exposure to dexamethasone (DEX) (*Trabecular Meshwork Inducible protein, TIGR*) [1]. It was independently discovered in the ciliary body [2] and in the normal retina [3]. The gene was later found to be expressed in non-ocular tissues, especially in heart and skeletal muscle [4]. However, MYOC retained special properties in the trabecular meshwork and its induction by DEX is specific to this tissue [5]. Soon after its discovery, mutations in the *MYOC* gene were found to be linked to 3–4% of primary open-angle glaucoma (POAG) [6] and to a large percent (10–30%) to juvenile open-angle glaucoma (JOAG), an early-onset and more severe form of the disease [7].

The glaucomas are a group of optic neuropathies caused by the degeneration and death of the retinal ganglion cells. In glaucoma, there is a progressive visual field loss and if left untreated, it leads to irreversible blindness. It is estimated that by 2020 there will be 79.6 million cases of glaucoma worldwide, with a high proportion of women and Asians [8]. POAG is the most common form of the disease, which in most cases, is triggered by an elevated intraocular pressure (IOP). In turn, elevated IOP is the result of an increased resistance of the trabecular meshwork tissue to the aqueous humor outflow.

To date, more than 70 *MYOC* mutations have been associated with glaucoma (http://myocilin.com/) [9]. Each of the mutations results in a slightly different phenotype, it is more prevalent in a given race and some have been speculated to be affected by environmental epigenetic factors (reviewed in [9]). Nevertheless, in every case, mutations in *MYOC* are associated with elevated IOP, ranging from mild to severe (http://myocilin.com/). Because of the relevance of this association, the MYOC protein has been extensively studied.

Myocilin is a 504 amino acid protein with a molecular weight of 55–57 kDa [10], [11]. The gene maps to 1q23–q24 [6] and contains three exons, which pretty much define three protein folding domains [4], [10]. The N-domain (aa 1 to 202) contains a signal peptide cleavage, a leucine zipper-like motif and is similar to the heavy chain of myosin [4], [10]. The C-terminal domain (aa 244 to 505), separated by a central linker (aa 203 to 205), is 40% homologous to olfactomedin, a major component of the extracellular matrix (ECM) of the olfactory neuroepithelium. The original finding that *MYOC* mutants mapped to the olfactomedin domain has held, and today, over 90% percent of pathogenic mutations are known to occur in that third exon of the protein (http://myocilin.com/).

Although considerable progress has been made, many questions regarding the function of wild-type MYOC and the molecular correlations of the different mutant variants to disease severity remain. Myocilin is processed and shed inside vesicles [12], [13]. In contrast to the wild-type, recombinant mutants in the olfactomedin domain, whether generic, glaucoma-associated, stop or missense, are unable to exit the cell in all cell types tried [5], [12], [14], [15]. Our earlier work also showed that mutant MYOC proteins lacking the olfactomedin domain are misfolded, form insoluble aggregates and accumulate in the endoplasmic reticulum (ER) [11]. Further, presence of increasing amounts of the recombinant mutant induces a fraction of the soluble, wild-type MYOC to move to the insoluble fraction and hamper its secretion [11]. Glaucoma-associated mutants are likewise insoluble [16] and hetero-oligomers with the wild-type are sequestered in the ER [17], leading to ER stress, activation of the unfolded protein response (UPR) and potential cytotoxicity [18], [19]. This data, supported by clinical findings on the absence of POAG in homozygous patients for certain MYOC mutants [20] led to the conclusion that MYOC-linked glaucoma was due to a gain of function.

In addition to glucocorticoids, *MYOC* expression is induced by a number of stress factors. Mechanical stretch, TGF-β, oxidative stress, heat shock and elevated IOP all induce *MYOC* in cells and tissues (review in [21], [22]. In addition, expression of *MYOC* mutants sensitizes cells to oxidative stress [23]. Myocilin interacts with several intracellular and extracellular matrix proteins (review in [24]). Recently it was shown to interact with components of the WNT signaling pathway [25], which was independently found to be associated to regulation of IOP [22], [26].

In the ER, MYOC undergoes an intracellular endoproteolytic cleavage in the central linker domain [27], [28]. This processing occurs in ∼40% of the wild-type protein and yields a 35 kDa fragment which is co-secreted with the full-length [28]. Myocilin mutants inhibit the proteolytic processing and the extent of inhibition has been correlated with the severity of the glaucoma phenotypes [28]. In addition, this proteolytic process modulates the molecular interactions of myocilin and reduces the formation of myocilin homoaggregates [29]. The enzyme responsible for this cleavage has been identified as Calpain II [30], a calcium-dependent cysteine protease present in most mammalian tissues.

All these findings put together reveal the potential involvement of many different genes in the functions leading to the *MYOC*-linked glaucoma. Previously, we reported a first microarray analysis using wild-type *MYOC* and high density oligonucleotide Affymetrix U133A GeneChips [31]. To now identify molecular differences among the effects of the causative glaucoma *MYOC* mutants, in this study we conducted an expression analysis on the transcriptome of primary human trabecular meshwork cells overexpressing *MYOC* mutants, and performed the analysis using the upgraded Affymetrix U133 Plus 2.0 GeneChips. We selected four representative mutants, based on different clinical outcomes, populations and/or relevance of the mutated codon. The Q368X variant is the most common (29% of the diseased causing variants) and results in a mild phenotype (http://myocilin.com/). Mutations R342K and D380N comprise 0.8% of the causing variants each, and are very severe, with a mean maximum IOP of 54 and 39 mmHg [32]. Both mutations have been reported only in one population, that of Ghana in West Africa. However while there is only one variant utilizing amino acid Arg342, the amino acid Asp380, highly conserved in all vertebrates, has produced four disease causing variants (D380N, D380H, D380A, D380G) [9], a recurrence known to occur very rarely in genetics. These four variants appear in different populations and in particular, the His and Ala changes result in intermediate glaucoma phenotypes and biochemical protein effects [15], [28], [33]. The last mutation, K423E, was selected because it occurs in two unrelated Caucasian populations [20], [34], has a severe clinical outcome and exhibits the interesting feature that homozygous patients do not manifest the disease [20]. A similar analysis recently published utilized transgenic flies and analyzed changes in the transcriptome of 2–3 day old insects’ whole heads [19].

Cystatin A (CSTA) is a member of the cystatin superfamily of proteins, some of which are active cysteine protease inhibitors, such as cystatin A (review in [35]). Within the cystatin superfamily, CSTA is characterized as a stefin [36]. Proteins of the stefin family, lack carbohydrates and disulfide bonds and have a molecular weight ∼11 kDa. This single chain protein forms tight complexes and inhibits the activity of papain-type proteases, cathepsin B, H and L [36], and presumably other intracellular cysteine protease inhibitors. The short N-terminal region of CSTA, and in particular the evolutionary conserved Gly-4 residue has been shown to play a key role in the binding of the CSTA inhibitor to the target proteases, papain, cathepsin B and L [37]. Mutations of Gly-4 to aminoacids with longer side chains like arginine were also shown to be more deleterious for the binding that mutations to alanine or serine which have small side chains [37]. Cystatin A is present in various tissues (epidermis, polymorphonuclear granulocytes, liver and spleen) and has also been found in extracellular fluids [35]. A loss of function mutation for CSTA was recently linked to two families of Middle Eastern origin exhibiting exfoliative ichthyosis, a scaly skin disease [38]. Cystatin A is a known myoepithelial cell marker and its downregulation plays a role in carcinogenesis, from breast to brain tumors [35]. It is believed that CSTA regulates cellular proliferation, tumor growth and metastasis. Cystatin A expression is a negative prognostic marker in breast tumors of lymph node negative patients [35]. Recently, levels of CSTA in serum, together with manganese superoxide dismutase and MMP2, were shown to be reliable biomarkers for the detection of nasopharyngeal carcinomas patients [39].

In the present study, we searched for genes and mechanisms affected by overexpression of four myocilin mutants in primary human trabecular meshwork cells. Using microarray profiles, we found that the myocilin mutants altered a high number of genes which had been previously associated with trabecular meshwork physiological and glaucomatous conditions. Some genes were shared by all mutants while some were mutant-specific. The extracellular matrix gene ontology (GO) category was the most enriched and most significant. Of the four most common mechanisms, genes in the UPR list were changed the most. More important, this study uncovered cystatin A, a cysteine protein inhibitor induced by all mutants, which reduced the processing of wild-type myocilin in vitro. These findings provide a molecular insight into mechanisms that trigger MYOC-glaucoma and raise the possibility of using silencing or inhibition of *CSTA* as a potential treatment of the MYOC-mutant development of glaucoma.

## Materials and Methods

### Generation of Adenoviruses Carrying Wild-type and MYOC Glaucoma-associated Mutants

**Figure 1:**
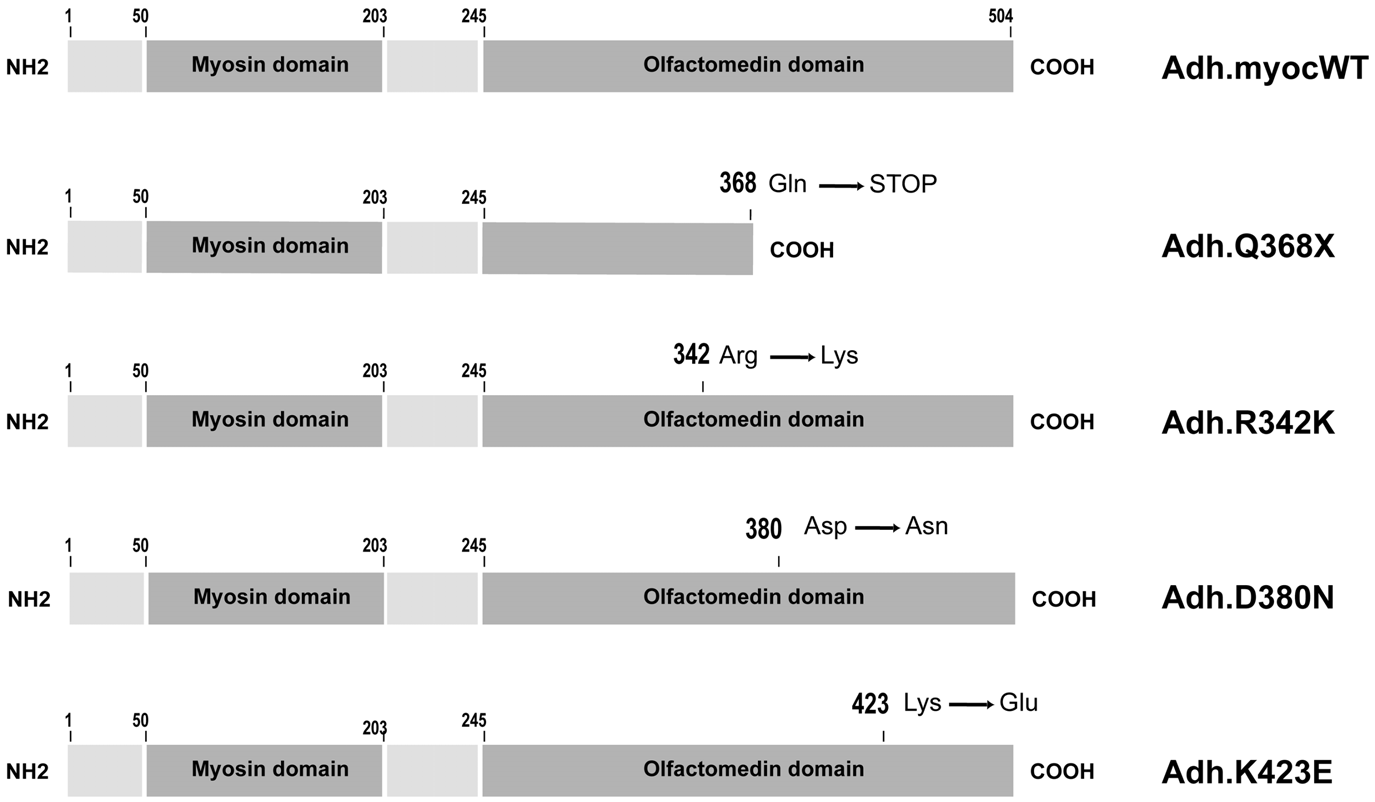
Schematic representation of MYOC wild-type and mutant proteins used for the adenoviral constructions. Myocilin protein contains a signal peptide cleavage (aa 1–50) and three folding domains. An N-terminal myosin domain (aa 50–203), a linker region (aa 203–245), and C-terminal olfactomedin domain (aa 245–504). All four selected mutants have mutations in the C-terminal olfaction domain. The Q368X mutation produces a truncated protein.

Plasmids carrying point mutations corresponding to four human *MYOC* mutations genetically linked to glaucoma were generated by site-directed mutagenesis using as a template clone pMC2 [11] and the QuickChange mutagenesis kit (Stratagene, La Jolla, CAL) [19]. The plasmids contained the MYOC mutations Q368X, R342K, D380N, and K423E (Figure 1). All mutant clones were re-amplified with 5′-*KpnI*-3′-*BamHI* ended primers to remove the *PmeI* site (*MYOC* nucleotides (nt) 4–1566) (all *MYOC* nt numbering is from access number AF001620) and subcloned into pCR2.1 (Invitrogen, Carlsbad, CA) for confirmation of sequence and presence of the mutations (clones pGL6, pGL9, pAC10, pAC14). *KpnI*-*NotI* restricted mutant cDNA fragments (1,596 nucleotides) were gel purified and inserted into pShuttle-CMV (generously donated by B. Vogelstein [40] for the generation of the recombinant adenoviral plasmid vectors (pGL7, pGL10, pAC11, pAC15). For the wild-type, the pKM1 plasmid (see below) was digested with *KpnI*-*NotI* and the isolated fragment inserted into the same pShuttle-CMV vector to yield pAC18 (total insert 1,601 bp containing 46 bp 5′ and 33 bp 3′plasmid sequences flanking the 1,522 *MYOC* wild-type coding region).

The shuttle vectors were then linearized with *PmeI* and electroporated into BJ5183-Ad1 cells for homologous recombination with an adenovirus backbone plasmid using the AdEasy Adenoviral Vector System (Stratagene) following manufacturer’s recommendations. The resultant Ad plasmid vectors (pGL8, pGL11, pAC12, pAC16, pAC19) were linearized with *PacI* and calcium phosphate-transfected (Clontech, Mountain View, CA) into early passages QBI-Human Embryonic Kidney (HEK) 293A cells (Qbiogene, Montreal, Quebec, Canada) for the production of the adenoviral recombinants (Adh.Q368X, Adh.R342K, Adh.D380N, Adh.K423E and Adh.MYOCWT). These viruses therefore carry each of the four *MYOC* mutants driven by the same CMV promoter. High titer stocks were obtained by propagation in the same QBI-HEK 293A cells and purification by double binding CsCl density centrifugation as described [41]. A control virus carrying the same promoter and no transgene (Ad5.CMV-Null) was purchased from Qbiogene (Montreal, Canada) and grown and purified in our laboratory. For Adh.Q368X, the virus particle number was determined by measurement of its optical density at 260 nm using the formula 1 µg of DNA = 2.2 X 10^10^ particles. For the remaining recombinants, physical particles were titered as viral genomes (vg)/ml as described [41] using a *MYOC* fluorescent TaqMan primers/probe Hs00165345_m1 (Applied Biosystems, ABI, Foster City, CA) and the *MYOC* plasmid pAC12 for the generation of the standard curve. Viral infectivity (infectious units [IFU]/ml) was measured with a rapid titer kit (AdenoX; Clontech) also as described [41]. Viral lots used in these studies had concentrations between 2 X 10^11^ and 1 X 10^12 ^vg/ml with infectivity values between 2 X 10^9^ and 5 X10^10^ IFU/ml A second set of viral stocks were grown at the University of North Carolina Vector Core facility. All viral stocks used in the experiments were checked for the overexpression of *MYOC* mRNA by TaqMan PCR (probe Hs00165345_m1) and MYOC protein by western blot (goat anti-human polyclonal, Santa Cruz Biotechnology #21243, Santa Cruz, CA)).

### Primary Culture of Human Trabecular Meshwork Cells

Primary human trabecular meshwork (HTM) HTM-72 and HTM-137 cell lines were generated respectively from the trabecular meshworks dissected from residual cornea rims of 29 and 39 years old donors (North Carolina Eye Bank) after surgical corneal transplants at the University of North Carolina Eye Clinic. The tissue was cut into small pieces, carefully attached to the bottom of the 2% gelatin-coated 35 mm dish, and covered slipped with a drop of MEM Richter’s Modification medium (IMEM, HyClone, Thermo Fisher Scientific, Waltham, MA) supplemented with 20% heat-inactivated fetal bovine serum (FBS, GIBCO catalog # 16140-071), 50 µg/ml gentamicin (Invitrogen). Cells from these specimens were not treated with enzymes and were allowed to grow from the explant for a period of 4 weeks changing the media every other day; upon confluency, cells were harvested and stored in liquid nitrogen. When reconstituted, these primary non-transformed cells are grown in complete medium consisting of IMEM, heat inactivated 10% FBS, gentamicin and subsist for seven to eight passages. In this study all cells were used at passage 4. These outflow pathway cultures comprise all cell types involved in maintaining resistance to flow. That includes cells from the three distinct regions of the trabecular meshwork plus cells lining the Schlemm’s canal. Because most of the cells in these cultures come from the trabecular meshwork, they are commonly referred to as “trabecular meshwork cells”.

### Delivery of Recombinant Adenoviruses to Primary Human Trabecular Meshwork Cells

Human trabecular meshwork primary cells at passage 4, seeded on either 3 cm or 10 cm dishes, were grown to between 65–90% confluency, washed twice with PBS and exposed to the recombinant adenoviruses (Adh.Q368X, Adh.R342K, Adh.D380N, Adh.K423E, Adh.MYOCWT and Ad5.CMV-Null) in 1 ml or 3 ml serum-free medium respectively. Multiplicity of infections (moi) ranged from 1.6 X10^3^–1.6 X10^4^ vg/cell and were randomly distributed among the replicas. After exposure to the virus for 90 min, complete media was added and incubation continued for 48 h or 72 h. Mutant-Null infections were always performed in the same day, but interspersed replicas expand over two years. Although FBS lot number was not recorded in each experiment, the same supplier, same catalog number of heat inactivated FBS was used in all experiments.

### RNA Extraction, Reverse Transcription and TaqMan-PCR Assays

Human trabecular meshwork cells cells were scraped from tissue culture dishes with guanidine thiocyanate buffer (RLT, Qiagen, Valencia, CA). Total RNA was extracted by loading the solution onto a QIA Shredder™ column (Qiagen) and continued by the use of the RNeasy Mini kit with on-column RNase-free DNAse digestion according to manufacturer’s recommendations (Qiagen). Purified RNA was eluted in 30 µl RNase-free water and the concentration measured with a NanoDrop ND-100 spectrophotometer (Thermo Fisher Scientific). Total RNA recoveries averaged 65.4±3.6 µg and 11.9±1.1 µg per 10 and 3 cm culture dishes respectively. RNA quality was assessed by measuring the size distribution on an Agilent Bioanalyzer (Agilent Technologies, Santa Clara, CA).

Reverse transcription (RT) reactions were conducted with 1 µg HTM cells RNA in a 20 µl total volume of proprietary RT buffer with RNAse inhibitor (High Capacity cDNA kit) (ABI) following manufacturer’s recommendations (25°C 10 min, 37°C 2 h, 85°C 5 min, then 4°C). Fluorescently labeled TaqMan probe/primers sets for human MYOC, CSTA, CXCL2, IGF1, MMP1, MMP3, MMP12, SFRP1, STC1, SNCA, RAB39B, THBD and 18S RNA were purchased from the ABI TaqMan Gene Expression Assays (ABI). The human probes used were: *MYOC* (Hs00165345_m1), *CSTA* (Hs00193257_m1), *CXCL2* (Hs00235956_m1), IGF1 (Hs00153126_m1), *MMP1* (Hs00233958_m1), *MMP3* (Hs00968305_m1), *MMP12* (Hs00899662_m1), *SFRP1* (Hs00610060_m1), *STC1* (Hs00174970_m1), SNCA (Hs01103383_m1), *RAB39B* (Hs00293395_m1) and *THBD* (Hs000264920_s1). All but one probe corresponded to sequences from different exons. The 18S RNA probe corresponded to sequences surrounding position nucleotide 609 (Hs99999901_s1). Reactions were performed in triplicate 20 µl aliquots using TaqMan Universal PCR Master mix No AmpErase UNG, run on an Applied Biosystems 7500 Real-Time PCR System, and analyzed by 7500 System SDS v.2.0.4 software (ABI). Relative Quantification (RQ) values between treated and untreated samples were calculated by the formula 2^−ΔΔC^_T_ where C_T_ is the cycle at threshold, ΔC_T_ is C_T_ of the assayed gene minus C_T_ of the endogenous control (18S), and ΔΔC_T_ is the ΔC_T_ of the normalized assayed gene in the treated sample minus the ΔC_T_ of the same gene in the untreated one (calibrator). Because of the high abundance of the 18S rRNA used as the endogenous control and in order to get a linear amplification, RT reactions from treated and untreated samples were diluted 10^4^ times prior to their hybridization to the 18S TaqMan probe. Statistical analysis was performed by the Student’s *t-test*.

### RNA Microarrays Hybridization

The RNAs from cells infected with *MYOC* mutants viral recombinants were prepared for hybridization to Human Genome U133 Plus 2.0 (n = 17) GeneChips (Affymetrix, Santa Clara, CA) at the University of North Carolina Functional Genomics Core Facility. These oligonucleotide microarrays contain 54,678 probe sets representing approximately 39,500 well-characterized human genes. The level of transcription of each gene represented on these chips is measured using the 11 nucleotide sequences which comprise each probe set. For the hybridization, total RNA (∼0.7 µg) was reverse transcribed into cDNA using a cDNA kit from Life Technologies with a T7-(dT)_24_ primer. Biotinylated cRNA was then generated from the cDNA reaction using the BioArray High Yield RNA Transcript Kit. The cRNA was then fragmented in fragmentation buffer (5X fragmentation buffer: 200 mM Tris-acetate, pH 8.1, 500 mM KOAc, 150 mM MgOAc) at 94°C for 35 min before the chip hybridization. 15 µg of fragmented cRNA was then added to a hybridization cocktail (0.05 µg/µl fragmented cRNA, 50 pM control oligonucleotide B2, *BioB*, *BioC*, *BioD*, and *cre* hybridization controls, 0.1 mg/ml herring sperm DNA, 0.5 mg/ml acetylated BSA, 100 mM MES, 1M [Na^+^], 20 mM EDTA, 0.01% Tween 20). 10 µg of cRNA was used for hybridization. Arrays were hybridized for 16 h at 45°C in the GeneChip Hybridization Oven 640. The arrays were washed and stained with R-phycoerythrin streptavidin in the GeneChip Fluidics Station 450. After this, arrays were scanned with the GeneChip Scanner 3000 7G Plus. Sample quality was assessed by examination of 3′ to 5′ intensity ratios of certain genes.

### GeneSpring Analyses

Row data CEL files from Affymetrix were imported into GeneSpring GX Expression Analysis software, version GS10 (Agilent Technologies). For analysis of expression changes between the *MYOC* mutants, *MYOC* wild-type and null infected samples, their files were pre-processed through the Robust Multichip Average (RMA) and replicas from each of the mutant, wild-type or control chips were grouped using the grouping feature of the program. An interpretation was created which identified the treated versus the control selecting average over replicates in each of the two conditions. To eliminate genes expressed at lower levels in at least one out of the conditions compared, normalized data were filtered by expression level using the signal intensity raw data at a lower cutoff value of 50.

To identify top-changers, data were subsequently filtered on Fold Change (FC) to select genes that exhibited at least a 1.5-fold increase or decrease in the MYOC infected cells. Variance of the samples was obtained by the unpaired *t*-test. Lists with genes altered FC ≥1.5 were generated in GS10 and exported to the hard drive. These original gene lists contain many annotations with no Entrez number, repeated annotations with the same Entrez gene, a few annotations recognizing more than one Entrez number (almost identical genes, often pseudogenes) as well as genes encoding hypothetical proteins and undefined open reading frames genes. Using standard excel sorting applications, we used a rational approach to clean and filter FC 1.5-fold gene lists for each of the redundant and the undefined genes parameters (see results below). Heat maps of the full range FC of the mutants on categories of genes from a given function (calcification, elastin collagen crosslinking, WNT signaling pathway, stress and UPR response and molecular signature of glaucoma) were created in GS v7.3. The custom made gene-function lists contained one Affymetrix ID per gene. In the case that one of the genes from the list was represented by more than one Affymetrix ID in the chip, we chose the ID which was more altered by the Q368X mutation. Overlapping Venn diagrams were created in GS10 with re-imported gene lists containing Entrez numbers (IDs with no Entrez removed). Gene ontologies of the overlapping gene list altered in all-mutants were created in GS v7.3. They were obtained from the GO SLIMS lists available in the GS7 software, which contain subsets of the terms in the whole GO.

### Microarray Data Submission

All microarray data is MIAME compliant and the raw data has been deposited in the ArrayExpress MIAME compliant database. The accession numbers of each of the five experiments along with login information are as follows:

Experiment name: MYOC.Q368XvsNull

ArrayExpress accession: **E-MEXP-3427.**

Username: Reviewer_E-MEXP-3427

Password: ebr8fswC

Experiment name: MYOC.R342KvsNull

ArrayExpress accession: **E-MEXP-3435.**

Username: Reviewer_E-MEXP-3435

Password: nzsut632

Experiment name: MYOC.D380NvsNull

ArrayExpress accession: **E-MEXP-3434.**

Username: Reviewer_E-MEXP-3434

Password: uuvvpXH4

Experiment name: MYOC.K423EvsNull

ArrayExpress accession: **E-MEXP-3439**

Username: Reviewer_E-MEXP-3439

Password: Ljwhbanh

Experiment name: MYOC.WTvsNull

ArrayExpress accession: **E-MEXP-3440**

Username: Reviewer_E-MEXP-3440

Password: kdXjkn3s

### Plasmids and Transfections

A human *MYOC* full coding recombinant expression vector was generated by amplifying pMC2 with primers 5′CACCTGCAATGAGGTTCTTCTGTG3’ (forward) and 5′TTTTCACATCTTGGAGAGCTTGAT3’ using Platinum Taq DNA Polymerase (Invitrogen) and cloning of the gel purified insert into pCDNA3.1D V5-His-TOPO (Invitrogen). The new plasmid, pKM1, contains 1522 bp (from -4ATG to TGA+3) of wild-type *MYOC* cDNA (19–1540 nt). A *MYOC*-V5 fused clone was generated by amplifying pKM1 with the same forward primer and a 5′TTTCATCTTGGAGAGCTTGATGTC3’ (reverse) which skips the stop codon (19–1534 nt) using high fidelity Advantage HD Polymerase Mix (Clontech). The gel purified fragment was incubated with pCDNA3.1D V5-His-TOPO to yield plasmid pMG29. A pCDNA3.1D V5-His-TOPO empty (CMV promoter, no transgene) was obtained during the sequence screening of above cloning procedures (pEmpty). All plasmids were transformed in TOP10 cells (Invitrogen) and confirmed by sequence. A TrueORFGold clone of human *CSTA* (stefin A) was obtained from Origene (Rockville, MD, cat.# RC203115). The pCSTA plasmid contains the full coding *CSTA* cDNA (nt 130–424 accession # NM_005213) fused to Myc-DDK tags. A TrueORFGold clone of human mutant *CSTA* (*CSTAm)* was designed and custom ordered to Origene. The *CSTAm* was obtained by site-directed mutagenesis of the codon GGA at nucleotide 10–12 of the ORF, coding for Glycine, to AGG coding for Arginine [37]. Change of the evolutionary conserved Gly-4 residue has been shown to decrease affinity of the binding of the CSTA inhibitor to Cathepsin B [37]. Plasmid DNAs were isolated either using either a Midi-Prep plasmid kit (QIAGEN) or a PowerPrep™ HP Plasmid Purification kit (Origene), which results in lower toxicity after the nucleofector transfection (unpublished).

Transfection of HEK293 cells was performed by the standard calcium phosphate method precipitating 7 µg of DNA (pMG29:pCSTA or pEmpty 1∶2.5) in 120 mM calcium phosphate (Clontech) per 3 cm dish, 40% confluent cells. After overnight exposure, cells were quickly washed with 1 mM EGTA/PBS, and incubated in IMEM 2% FBS for an additional 48 h. A change of medium was done 24 h before harvesting. Transfection of primary HTM cells was performed using nucleofector technology (Amaxa Lonza, Basel, Switzerland), their basic kit for Primary Mammalian Endothelial Cells and the protocol previously described [42]. Briefly, cells were split 24 h before transfection, trypsinized, counted, and centrifuged at 100 g for 10 min. Cell pellets were resuspended in the proprietary mammalian endothelial solution at a concentration of 4 X 10^5^ cells/100 µl. Plasmid DNA encoding the V5-fused MYOC protein (pMG29) was mixed with that of plasmids encoding either the DDK-fused *CSTA* (pCSTA) or an empty transgene (pEmpty) and added to the cells at a total of 3 µg (pMG29:pCSTA or pEmpty 1∶2). Cells-DNA solution was electroporated on the nucleofector apparatus (Amaxa Lonza) using program T-23 and allowed to recover for 15 min in pre-warmed serum-containing media inside the CO_2_ incubator. Following the recovery period, cells were gently transferred to warm medium-containing 3 cm dishes. After 24 h, media was changed with IMEM 10% serum followed by replacement with serum-free media 8 h later. Cells were then maintained for an additional 24 h (48 h post transfection) before being harvested for the extraction of proteins.

### Western-Blots and Antibodies

Proteins extracted from transfected HEK293 and HTM primary cells were assayed for levels of the different forms of the MYOC protein. Cultured media was collected and saved. Adhered cells were washed 2X with cold PBS and scraped from the dish with 100 µl of modified RIPA buffer containing 1X protease inhibitor cocktail (Roche Applied Science, Indianapolis, IN). Aliquots of 4 µl from either the cell lysates or the media were mixed (1∶2 vol) with Laemmli buffer (Bio-Rad, Hercules, CA) containing 5% β-mercaptoethanol and loaded onto 4–15% SDS-PAGE Tris-HCl polyacrylamide gels (Bio-Rad). After running, gels were electro-transferred to PVDF membranes (Bio-Rad), blocked with 5% nonfat dry milk (Bio-Rad) in PBS-0.2% Tween 20 (Sigma-Aldrich, Saint Louis, MO) for 2–5 h and incubated at 4°C for 3 h to overnight with anti-V5 mouse monoclonal antibody (Invitrogen) (1∶400). Primary antibody reaction was followed by incubation with anti-mouse IgG secondary antibodies conjugated to horseradish peroxidase (1∶2000; Pierce Biotechnology, Rockford, IL) for 1 h at room temperature. Immunoreactive bands were visualized by chemiluminescence ECL Plus western blotting detection system (GE Healthcare Biosciences, Piscataway, NJ) and membranes were exposed to X-ray film (BioMax MR; Kodak, Rochester, NY). For controls, blots were re-probed with a monoclonal anti β-actin (synthetic peptide) (Sigma) (1∶5000) and a monoclonal anti-DDK (synthetic peptide) (1∶200) (Origene) for 1 h at room temperature and overnight at 4°C, followed by anti-mouse IgG secondary antibodies conjugated to horseradish peroxidase (1∶5000; Pierce Biotechnology). Full length and processed MYOC bands were captured using a Chemi System equipped with a GelCam 310 camera, PCI digitizing image acquisition board, EpiChemi II Darkroom with transilluminator and VisonWorksLS image acquisition software v.7.0.1 (UVP, Upland, CA). Densitometry of each band was performed with the provided software to obtain mean intensity values (average of intensities of all pixels of band region, minus average intensity of the background pixels). Default background is equal to the sum total of the perimeter around each band region, three pixels wide. The percentage of the processed band in each treatment was calculated by dividing its mean density by the sum of the mean density of the process plus unprocessed bands.

## Results

### Adenoviral Vectors Carrying four *MYOC* Mutants Linked to Glaucoma

The adenoviral vectors were constructed as indicated in methods and the *MYOC* mutant proteins encoded by their inserted cassettes are shown in Figure 1. Prior to the generation of RNA to hybridize to the Affymetrix chips, the mutants vectors were tested on HTM cells for the overexpression of *MYOC* RNA and proteins using TaqMan PCR and WB as described above. Upon normalization to 18S, the levels of MYOC RNA on cells infected with each of the mutants over the levels of MYOC RNA on cells infected with Ad.Null increased significantly for all mutants. Infection with Adh.Q368X produced the truncated form of the protein while Adh.R342K, Adh.D380N and Adh.K423E produced the same size protein as the wild-type. Although the TaqMan mRNA levels produced by Adh.K423E mutant were similar to those of the other mutants, the levels of its produced protein were the lowest, indicating either a higher susceptibility to degradation of this MYOC mutant protein, or a lower recognition by the antibody due to faulty folding.

### Number of Genes Altered in HTM Cells Overexpressing each of the Four MYOC Mutants

To gain a first insight into the extent of changes occurring in the transcriptome of HTM cells overexpressing *MYOC* mutants we counted the number of genes altered between the mutants and controls cells (cells infected with Ad5.CMV-Null viruses). To further understand the differences caused by the mutants and the wild-type, we re-analyzed the comparisons using the chips of the *MYOC* mutants versus those of the *MYOC* wild-type. To override the potential differences between primary cell lines derived from different individuals, all overexpression experiments were conducted in the same cell line at the same passage (HTM-72, passage 4).

All comparisons were done using non-redundant lists of overexpressed genes. For this, the gene lists generated in Gene Spring were cleaned according to the following criteria. Affymetrix IDs that did not have an Entrez number were removed. In the few cases where one Affymetrix probe set ID number recognized more than one gene (usually almost identical genes or pseudogenes), one of them was selected. In the cases of redundant Entrez numbers, that is, when there were several Affymetrix IDs for the same Entrez number, we selected the ID that had been most altered.

**Figure 2:**
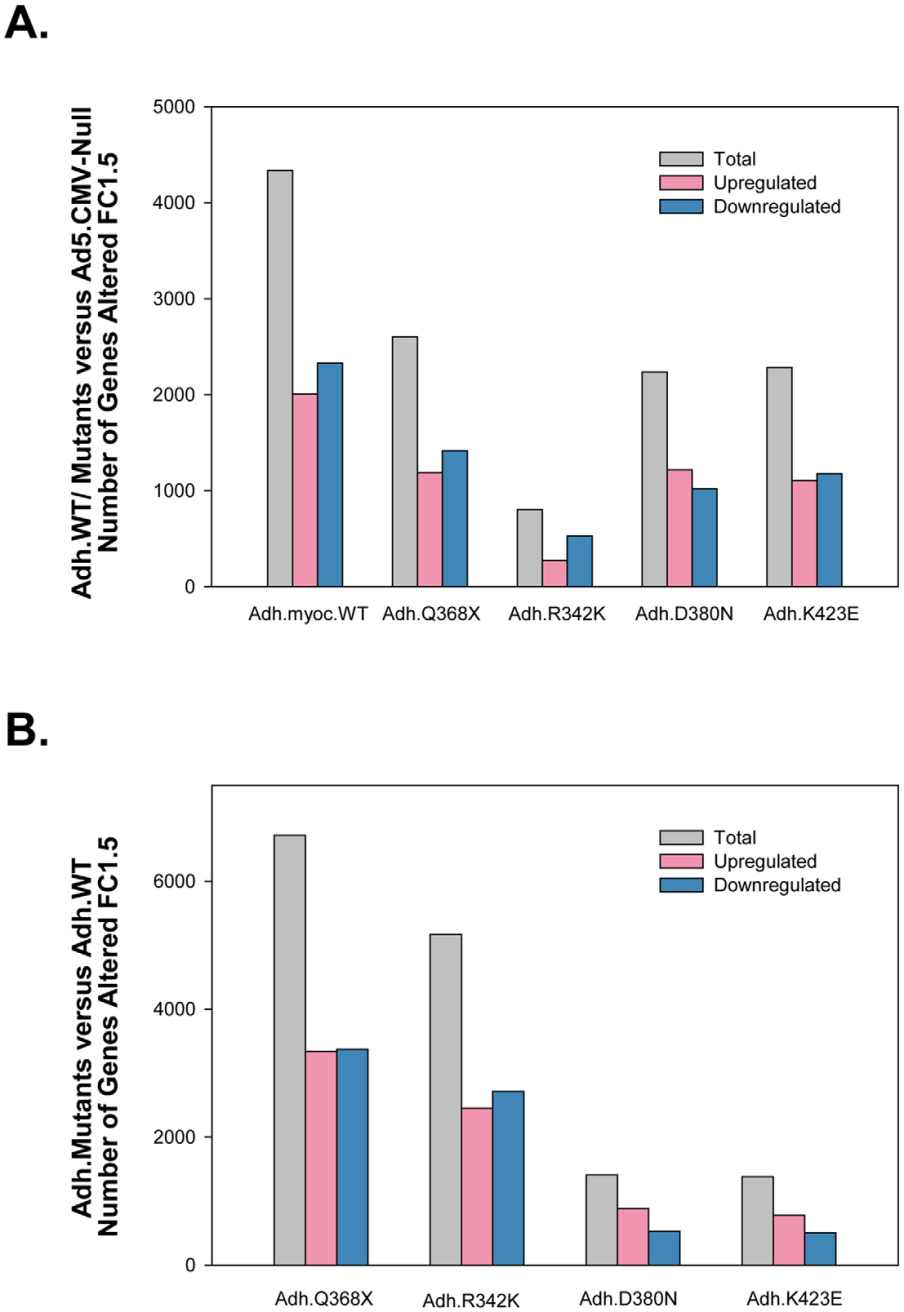
Wild-type *MYOC* and different *MYOC* mutants alter different gene numbers on the trabecular meshwork transcriptome. Adenoviral vectors carrying either wild type, four *MYOC* mutations cassettes or no transgene (Ad5.CMV-Null) were infected on primary human trabecular meshwork cell line HTM-72 to overexpress MYOC proteins. The expression of genes in the wild-type or mutant-treated cells was obtained using Affymetrix GeneChips (n = 17). GeneSpring 10 and Excel software were used to generate non-redundant gene lists with cutoff fold-change values of ≥ and ≤ 1.5. A: number of genes altered in cells treated with the wild-type or each of the mutants compared with the number altered in cells treated with the empty virus (Adh.WT/Mutants versus Ad5.CMV-Null). B: number of genes altered in cells treated with the each of the mutants compared with the number altered in cells treated with the wild type virus (Adh.Mutants versus Adh.WT).

Using replicas for each of the mutants and for the controls (Adh.MYOC n = 10, 2 per construct; Ad.CMV-Null n = 7) and selecting the methodology and filters outlined above, we generated lists of 1.5-fold altered genes. Out of the 54,678 spots in the array, the total number of non-redundant genes altered ≥1.5X was higher in the wild-type than in each of the mutants (Figure 2A). Overexpression of the wild-type induced the change of 4,337 genes while that of the Q368X mutation, which was the second highest, altered 2,603 genes. While the number of genes altered in the D380N (2,237) and K423E (2,283) mutations were pretty similar to those changed by the stop mutation, the mutation R342K induced only 803 changes in the HTM transcriptome (Figure 2A). From the total number of altered genes, the number of up- and downregulated seemed to be similar in wild-type and mutants with approximately one-half of the total altered in each category. The highest difference was observed in the R342K mutant, which had a higher number of downregulated genes (274 genes up- and 529 genes downregulated) (Figure 2A).

When comparing the number of genes altered in the mutants with those altered in the wild-type, we found that the stop mutation Q386X had the highest difference with a total of 6,716 (3,342 up and 3,374 down) (Figure 2B). Overall, looking at this parameter, it appeared that the mutants fell into two groups with considerably different patterns. The mutations Q368X and R342K had similar gene number changes and were much higher than those of D380N and K423E which in turn were similar to each other (1,411 and 1,384). Together this result on the absolute number of altered genes indicates that different *MYOC* mutants can alter the HTM transcriptome to a different extent and could therefore have a different severity outcome on the function of the tissue.

### Selected Top-changers Trabecular Meshwork Relevant (TMR) Genes from MYOC Mutants Induced Lists

The 100 top-changers (50 upregulated and 50 downregulated) (p≤0.05) from each of the mutants compared to Ad5.CMV-Null are submitted as supporting material (Tables S1, S2, S3, and S4). To generate these lists we started with the non-redundant lists obtained above and rationally eliminated those ID numbers with annotations for LOC hypothetical proteins, FAM (family w/sequence similarity), noncoding RNAs and pseudogenes. Myocilin was the most upregulated gene in all lists except in that of the K423E mutant, serving as a control of the overexpression. Levels of overexpressed *MYOC* were identical after infections with Q368X and R342K, and about half after D380N, though still at the top of the list (Tables S1, S2, S3, S4, S5, and S6). TaqMan PCR confirmed that the K423E mutant was highly overexpressing *MYOC*, so its absence from the top gene in the array list was interpreted as a low efficiency of this mutant’s cDNA to hybridize to the *MYOC* Affymetrix gene chip spot.

**Figure 3:**
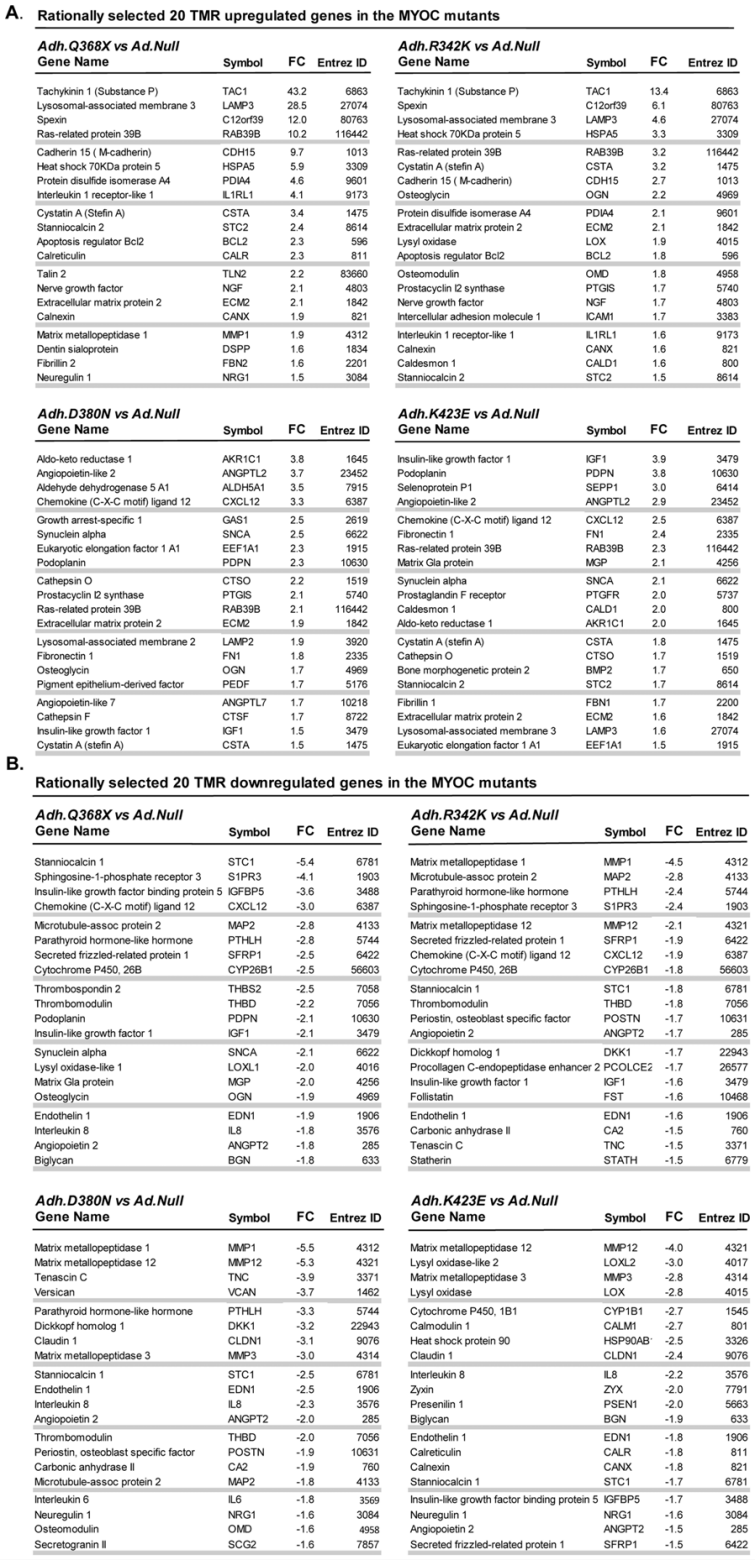
*MYOC* mutants’ top-changers contained numerous human trabecular meshwork relevant genes. Adenoviral vectors carrying four *MYOC* mutations cassettes and no transgene (Ad5.CMV-Null) were infected on primary human trabecular meshwork cell line HTM-72 to overexpress MYOC mutant proteins. The expression of genes in the mutant-treated cells was compared with that of the cells treated with the empty virus, using Affymetrix GeneChips (n = 15). Non-redundant gene lists from the cutoff FC value of ≥ and **≤** 1.5 of each mutant were screened for trabecular meshwork relevant (TMR) genes. Each selected TMR gene was manually cross-checked to identify its expression in the other three mutants. A: twenty selected upregulated TMR genes in Q368X, R342K, D380N and K423E. B: twenty selected downregulated genes in Q368X, R342K, D380N and K423E.

To further get an insight into the extent of how many of those *MYOC* mutant-induced genes were encoding trabecular meshwork functions, and to investigate whether there was sharing of genes among the four mutants, we screened each of the FC 1.5 lists for TMR. For this analysis, we separated the up- and downregulated genes of each mutant list, sort them by FC and, without taking into account the microarray p-values, scrolled down to rationally select 20 genes from each direction with TM and/or glaucoma-related functions (TMR) (total 160 genes). Then, we performed a manual cross-check and identified whether each of the selected genes in each mutant was present in the other three (Figure 3A and 3B).

Overall, we had a total of 75 unique TMR genes altered in any of four *MYOC* mutants, many of which had previously been reported as responders to glaucomatous insults in independent studies. Eight genes were altered in all the mutants. *Ras-related protein 39B (RAB39B), Cystatin A (CSTA)* and *Extracellular matrix protein 2 (ECM2)* were upregulated, while *Endothelin 1* (*EDN1*), *Angiopoietin 2* (*ANGPT2)* and *Stanniocalcin 1* (*STC1*) were downregulated. *Chemokine* (C-X-C) *ligand 12* (*CXCL12*) *and Insulin-like growth factor 1 (IGF1, Somatomedin C)* showed different regulation depending on the mutants.

The upregulated TMR genes induced by the Q368X mutant appeared to be more similar to those induced by R342K and different from those induced by D380N and K423E. Thus, including the all-common genes, 14 out of the 20 TMRs (70%) in Q368X were shared by R342K while only 6 and 7 in each of these were shared by either D380N and/or K432E respectively. Interestingly, upregulated D380N and K423E lists shared a higher similarity between themselves (12 out of 20, 60%).

The dowregulated TMR genes induced by the Q368X, were however very similar in the four mutants. Thirteen out of 20 downregulated TMR in Q368X were altered in R342K and all genes but one, *Lysyl oxidase-like 1* (*LOXL1*), were altered in D380N and K423E. *LOXL1*, a gene recently linked to pseudoexfoliation glaucoma [43] was altered only by the Q368X mutation.

Among the altered genes shared by Q368X and R342K (mutant set #1) and not by D380N and K423E (mutant set #2), were *Tachykinin 1 (TAC1)*, *Protein disulfide isomerase A4 (PDIA4), Cadherin 15* (*CDH15), Apoptosis regulator BCL2 (BCL2)* and *CYP26B1*. The *TAC1* gene, encoding the precursor of neuropeptide substance P, was previously identified as a mechanosensitive gene in the human trabecular meshwork intact tissue [22], [44]. Protein disulfide isomerase A4, which plays a key role in protein folding, had been found to be altered by TGFβ2, DEX and by elevated IOP in the trabecular meshwork tissue during the homeostatic response period [44]–[46].

Among the genes altered by set #2 and not by set #1 mutants we found Aldo-ketoreductase 1 (AKR1C1), Angiopoietin-like 2 (ANGPTL2), Fibronectin 1(FN1), Matrix metallopeptidase 3 (MMP3) and α-Synuclein (SNCA), which affect various functions, such steroid metabolism, inflammatory signaling and ECM organization. The synucleins are proteins highly expressed in the brain and are involved in presynaptic signaling and membrane trafficking.

Two of the all-common genes (*IGF1* and *CXCL12*) support the notion that mutant’s set #1 had similar effects on the trabecular meshwork transcriptome, which were different from those of set #2. Expression of *IGF1* and *CXCL12* was down in set #1 and up in set #2. Insulin growth factor 1 was classified as an individual responder to elevated IOP in human perfused organ cultures and pressure [22] while *CXCL12* is a member of the same chemokine family as CXCL2, a general responder to elevated IOP. Another case where *MYOC* mutants had opposite effects on a given gene was that of *Podoplanin* (*PDPN*), a lymphatic marker regulated by elevated IOP. Podoplanin is upregulated by D380N and K423E while is downregulated by Q368X.

There were only a few TMR genes that were altered by just one mutant. These were: *Lysyl oxidase-like 1* (*LOXL1*) (downregulated by Q368X), *Procollagen C-endopeptidase enhancer 2* (*PCOLCE2*) (downregulated by R342K), *Pigment epithelium-derived factor* (*PEDF*) (downreguled by D380N), *Lysyl oxidase-like 2* (*LOXL2*) and *Cytochrome P450 1B1* (*CYP1B1*) (downregulated by K423E), *and Bone morphogenetic protein 2 (BMP2*) and *Fibrillin 1 (FBN1)* (upregulated by K423E). Curiously, three of these genes have been genetically linked to glaucoma [43], [47], [48], suggesting an additional physiological link of the mutants with glaucoma at the molecular level.

Another trabecular meshwork gene previously known to be altered under other insults (IOP and DEX) was *Thrombomodulin (THBD)* [45]. Thrombomodulin is a vascular endothelial cell receptor that binds to thrombin and is involved in the inhibition of blood clotting. It has been speculated that *THBD* plays a role in maintaining the fluidity of the aqueous humor [45]. In this study, *THBD* was downregulated in all but the K423 mutant, suggesting a potential detrimental effect caused by the *MYOC* mutants. The same occurred with insult-altered metallopeptidases *Matrix metallopeptidase 3* (*MMP3*) (*Stromelysin* 1) [49] and *Matrix metallopeptidase 12* (*MMP12*) (*Macrophage elastase*) [22] which were shown downregulated here by the *MYOC* mutants suggesting that they would contribute to a decrease outflow.

Although many of the rationally selected TMR genes had significant microarray FC values, some of them had not. Thus, a representative sample of ten genes with no significant microarray p-values in at least one mutant were analyzed in triplicate by the more rigorous TaqMan PCR assay in a different primary cell line. HTM-137 and HTM-134 lines were infected with Adh.Q368X, Adh.R342K, Adh.D380N, Adh.K423E plus the control Ad.Null viruses, their RNA extracted at 48 h post-infection and reversed transcribed. Results are included in Table S5. Although not in every case the absolute TaqMan FC alteration value was similar to that of the microarray data (different individual, different culture and different viral stock), only four of the forty TaqMan assays performed showed a p-value higher than 0.05. Of the four (MMP1 and SFRP1 in Adh.Q368X and MMP12 and SFRP1 in Adh.K423E), two of them had a p<0.05 in the microarrays (Table S5). The remaining of the genes exhibited highly significant p-values in all mutants (Table S5).

Altogether these results indicate that overexpression of *MYOC* mutants share many elicited changes with other known glaucomatous insults. Further, they indicate that distinct *MYOC* mutants could have specific effects on the trabecular meshwork cells transcriptome.

### Pattern of Expression of Trabecular Meshwork Relevant Functions (TMR.F) Gene Lists in the Four MYOC Mutants

**Figure 4:**
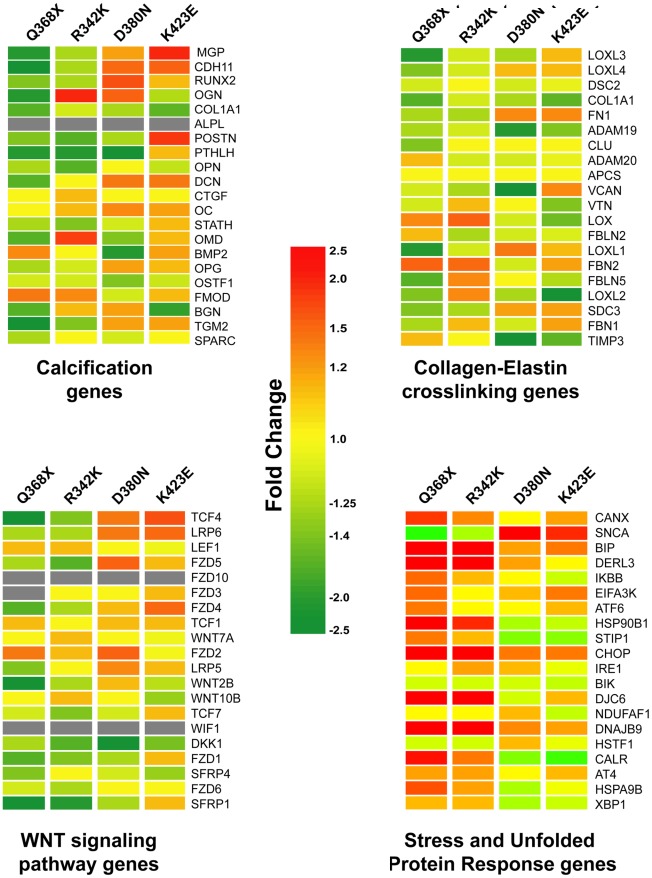
*MYOC* mutants induced changes on genes of trabecular meshwork relevant functions, especially on the UPR. Heat maps of set of genes representing four relevant trabecular meshwork functions. The four categories gene lists were costumed generated by literature review. Each row represents the fold change (Adeno.MYOC mutant over Adeno.Null) for a single gene in each of the four mutants. Each column represents the fold changes for all genes of the category in one *MYOC* mutant. The fold change for each gene is visually represented by a color, which is given by the scale bar in the center of the figure. Heat maps were generated with genes lists containing the full range of fold changes. Gray cells indicate that the expression of the giving gene was below the signal intensity cutoff value and was considered absent.

Next we investigated the overall expression pattern of set of genes known to be associated with selected TMR.F. In other words, we wanted to know whether genes involved in calcification mechanisms [50], collagen-elastin cross-linking functions [51], the WNT signaling pathway [22], [25], [26] and stress response had been affected by overexpression of *MYOC* mutants. We custom made comprehensive gene lists of the four mechanisms each containing 20–21 genes, and ran heat maps against the complete FC lists of each of the *MYOC* mutants (Figure 4). Each row of the map represents the response of a single gene to the overexpression of each of the four *MYOC* mutants, expressed in FC of the *MYOC* mutant- versus Ad5.CMV-Null infected cells. Each column represents the set of genes selected per category. The range of the FCs of the genes in the calcification, collagen-elastin and WNT maps by all mutants was between +2.1- and −3.3-fold. The range of FC of the stress and UPR genes was much larger, between +8.5 and −2.0-fold. Altered *MYOC* mutant genes which did not pass the minimal expression criteria (absent signals) have no color and appear as gray-cells. Overall, most of the genes of in each of the four functional categories were up or down regulated by at least one of the *MYOC* mutants and only a few were not affected in any mutant (represented by yellow colors). The trend of the similarities of the mutants in set #1 distinct from those in set #2 can be also seen here, mostly on genes with highest or lowest changes.

#### Calcification genes

The mutation Q368X downregulated most of the genes in the calcification category, including *Matrix Gla* (*MGP*), an inhibitor of calcification and one of the ten most abundant genes in the trabecular meshwork [52], [53]. Concurrently, Q368X upregulated *BMP2*, an inducer of calcification and bone formation which loses its activity upon binding to the inhibitor *MGP* [54]. These two changes together would suggest that the Q368X mutant is prone to induce calcification in the HTM cells which in turn would provoke hardening of the tissue and reduce outflow facility. In contrast, the same *MGP* gene was upregulated in the set #2 mutants, where *BMP2* was moderately reduced, indicating a different degree of involvement of this pathway by different *MYOC* mutants. The *Connective Tissue Growth Factor* (*CTGF*) gene, which has been extensively studied for its relevance in ECM deposition in trabecular meshwork function [55] was the less altered in all mutants, and *Collagen type 1 alpha 1* (*COL1A1)*, an important structural component of the trabecular meshwork ECM, was downregulated in the four mutants assayed. Genes like *Osteomodulin* (*OMD*) and *Osteoglycin* (*OGN*, also known as *Mimecan*), members of the SLPRs family of proteoglycans were markedly upregulated only by mutant R342K and appeared downregulated by Q368X. These two genes are markers of osteoblast differentiation [56], and respond to mechanical stress [44], [46]; *OGN* was found upregulated in tissues from POAG patients [57]. Another gene, *Periostin* (*POSTN*, also known as *Osteoblast- specific factor2*), secreted by osteoblasts, was highly upregulated only in K423E. Periostin was previously shown to be upregulated by the glaucomatous insults of mechanical strain and TGFβ2 [58], [59]. Lastly, *Transglutaminase2* (*TGM2*), which catalyzes the cross-linking of numerous ECM proteins, whose presence in vascular smooth muscle cells (VSMC) is key to mineralize their matrix and which is present in the aqueous humor of glaucomatous patients [60] was very much downregulated in Q368X (Figure 4 upper left panel).

#### Collagen-Elastin Crosslinking

The most upregulated genes in this TMR.F category were *Fibrillin 2* (*FBN2*) and to a lesser extent *Lysyl oxidase* (*LOX*). They were induced with different intensities in mutant’s set #1 Q368X and R342K. Lysyl oxidase is an enzyme involved in post-translational modifications of collagen and elastin and in the formation of intra/intermolecular cross-links, and *FBN2* is involved elastic fiber assembly. Lysyl oxidase-like 1, which is linked to Pseudoexfoliation (PEX) glaucoma, and which is heavily bound to the PEX material of PEX patients was very downregulated in Q368X, while other two PEX relevant components [51], *FBN1 and Fibulin 5* (*FBLN5*), were slightly altered in all four mutants. The mutation D380N most dowregulated *Versican* (*VCAN*), another PEX component and mechanical strain regulated gene [44], and two metalloproteinases/inhibitors, *ADAM metallopeptidase 20* (*ADAM20*) and *Tissue inhibitor of metalloproteinases 3* (*TIMP3*), which are involved in maintaining the balance of the ECM and therefore affecting the elastin network. Overall it seemed that the effect of the four *MYOC* mutants had subtle effects on genes involved in the formation of the elastin network and that such light effect was mostly that of downregulation (Figure 4 right panel).

#### WNT signaling

Genes involved in this pathway were downregulated preferentially by mutant’s set #1. Mutant Q368X most downregulated *Secreted frizzled-related protein 1 (SFPR-1),* an IOP responder gene [22] which is present in glaucomatous trabecular meshwork cells and causes elevated pressure [26]. The most differently regulated gene between set #1 and set #2 was *Transcription factor 4* (*TCF4*), which interacts with β-catenin and mediates transcription of WNT targeted genes. The upregulation of *TCF4* in D380N and K423E could be an indication that these two *MYOC* mutants would utilize more the WNT pathway to induce transcription than the other two. In contrast, *Dickkopf-1* (*DKK-1*), an antagonist that prevents activation the WNT pathway, was downregulated by all mutants, especially by D380N. Two of the genes of this pathway, the intronless transmembrane receptor *Frizzled family receptor 10* (*FZD10*), and the WNT protein inhibitor *WNT inhibitory factor 1* (*WIF1*), were not expressed in these set of trabecular meshwork cells (Figure 4, lower left panel).

#### Stress and Unfolded Protein Response (UPR)

Because *MYOC* mutants are known to accumulate in the ER and thus affect the UPR [11], [18], [19], [23], we next examined the expression of twenty genes encoding the most common stress proteins (Figure 4, lower right panel). We found that expression of genes in the stress category was considerably affected by the *MYOC* mutants. Furthermore, expression of these genes was clearly different in set #1 and #2 mutants. The mutant having a major effect on this TMR.F was the stop mutation Q368X. In this mutant, the expression of all but two of the selected most commonly associated proteins were markedly altered. Among them the canonical UPR proteins *Bone-inducing protein* (*BIP*), *DAN-damage-inducible transcript 3* (*CHOP*) and *Calreticulin* (*CALR*) were markedly upregulated, as well as chaperones *Der1-like protein 3* (*DERL3*) and heat shock proteins *HSP90B1, DNAJB9 and DJC6*. Interestingly, *CALR*, an ER Ca^+^ binding protein that binds to the glucocorticoid receptor, together with *Stress-induced-phosphoprotein 1* (*STIP1*) was one of the most down regulated protein in the set #2 mutants. Another protein in which the direction of expression was clearly reversed in both sets was *SNCA*, an important component of the amyloid plaques in Alzheimer patients. *SNCA* was the most UPR upregulated transcript in D380N and K423E and the most downregulated in Q368X and R342K. Except for the *NADH dehydrogenase (ubiquinone) 1 alpha subcomplex, assembly factor 1* (*NDUFAF1*), the UPR proteins altered in transgenic flies carrying these mutants [19] are also altered in the human trabecular meshwork tissue, validating the fly system as a good genetic model of glaucoma.

### Pattern of Expression of the Physiological Trabecular Meshwork Biomarkers Gene List in the Overexpressed Four *MYOC* Mutants

**Figure 5:**
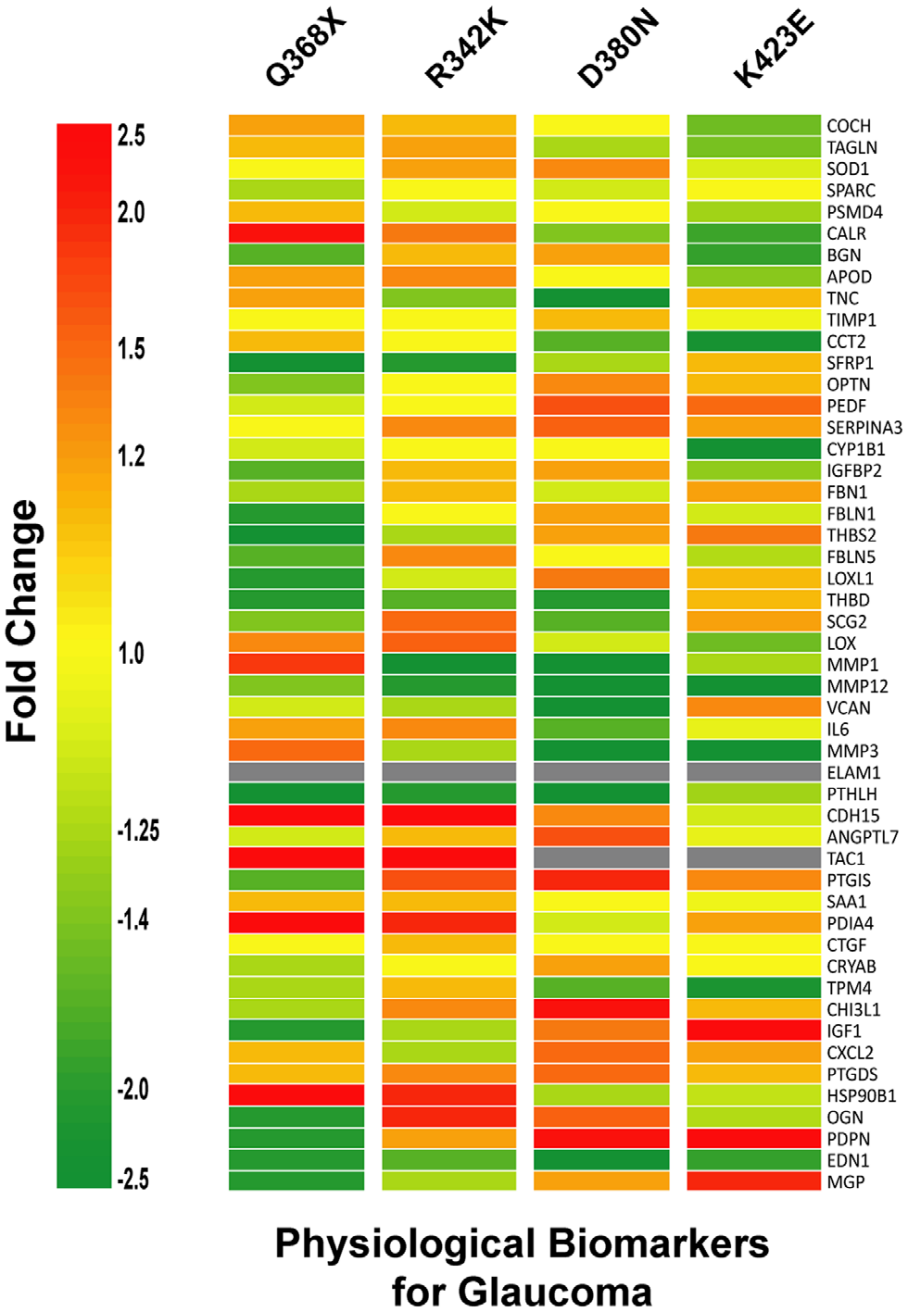
*MYOC* mutants altered most physiological biomarkers of glaucoma. Heat maps of a gene list containing 50 potential biomarkers for the human trabecular meshwork (Table S6). The biomarker gene list was generated by a comprehensive review of trabecular meshwork expression studies as indicated in the result section. Each row represents the fold change (Adeno.MYOC mutant over Adeno.Null) for a single gene in each of the four mutants. Each column represents the fold changes for all biomarkers in one *MYOC* mutant. The fold change for each gene is visually represented by a color, which is given by the scale bar at left. Heat maps were generated with genes lists containing the full range of fold changes. Gray cells indicate that the expression of the giving gene was below the cutoff of signal intensity value and was considered absent.

We next were interested to determine the ability of the *MYOC* mutants to alter a set of genes potentially associated with glaucoma and or glaucomatous insult. The 50 gene list is based on a previously published potential molecular signature of glaucoma genes [45]. It is also based on a more recent list of physiological biomarkers of glaucoma built on a revision of trabecular meshwork gene expression studies from us and other investigators (Table S6). The results of the heat map showing the expression of the 50 genes in all the mutants are shown in Figure 5. All genes except *Endothelial adhesion molecule 1* (*ELAM1*) were present in all the mutants, indicating that *ELAM1* did not pass the filter set up for analysis of expression in the HTM-72 cell line. Because expression of this gene has been detected in whole trabecular meshwork tissues [22], its absence here indicates a marked downregulation of the gene when the cells are set up in culture.

Although some of the genes were expressed in set #1 differently than in set #2, the overall patterns of the different sets was not as marked as seen in the other gene lists categories. It is interesting to observe that 78% of the genes from this independent gene list were shown to be altered more ≥1.5X in at least one of the four *MYOC* mutants (Tables S1, S2, S3, S4, and Figure 3A and Figure 3B), an indication of the physiological causative role of *MYOC* in glaucoma. Very few genes were altered in the same direction in all four mutants. Two of the three downregulated genes, *EDN1* and *MMP12*, had been mentioned above. The third one, *Parathyroid hormone-like hormone* (*PTHLH*), is a protein that regulates vascular calcification [61] and that has previously been shown to be induced by DEX in the trabecular meshwork [62]. Only the gene encoding *Prostaglandin D2 synthase* (*PTGDS*), an enzyme that catalyzes the conversion of PGH2 to PGD2, was lightly upregulated in the four mutants.

Overall, a similar number of genes were up- and downregulated in mutants Q368X, D380N and K423E while R342K showed a lower number of downregulated genes. However, alteration of specific genes varied. The genes most altered by Q368X were *TAC1*, *CDH15*, *PDIA4* and *HSP90B1* (upregulated 43X, 9.7X, 4.6X and 3.3X respectively), and *PTHLH*, *SFRP1* and *Thrombospondin2* (*THBS2*) (all downregulated −2.5X). The mutant R342K shared the four most upregulated and two of the most downregulated (*PTHLH*, *SFRP1*) with Q368X. Interestingly, the gene most downregulated by R342K, *MMP1* (−4.5X), was shared with the D380N and K423E mutants but not with Q368X, where *MMP1* was upregulated. Mutants D380N and K423E shared *PDPN* among their three top upregulated and the metallopeptidases *MMP3* and *MMP12* among the top downregulated. Another interesting difference among the effect of the four mutants on the transcriptome was the total lack of expression of *TAC1* (precursor of the substance P neuropeptide) in D380N and K423E, while it was the most induced gene in Q368X and R342K. Mutant Q368X was the only one to exhibit genes with unique responses, that is, with changes in expression that were opposite to the changes incurred by the other three (*MMP1*, *MMP3* and *Prostacyclin synthase, PTGIS*).

Of the four genes induced by the highest number of glaucomatous insults, *Angiopoietin-like 7* (*ANGPTL7), PDIA4, Superoxide dismutase 1* (*SOD1*) *and Tropomysin 4* (*TPM4*) (Table S6), only *PDIA4 (*upregulated in Q368X and R342K), and *TPM4* (downregulated in K423E) were markedly altered in some of the mutants. *PDIA4* is an ER protein with isomerase activity on S-S bonds, and *TPM4* is actin binding protein involved in the contractile system of the cell.

Altogether, these different and common influences on the transcriptome triggered by the four mutants reveal a potential molecular reason as to why each of the *MYOC* mutants have a distinct clinical outcome and affect a particular group of the population.

### Genes Altered in All Mutants

In addition to finding genes by which each of the mutants may exert their damage, we were interested in searching for a common altered gene whose mechanism of action could potentially be applied to all *MYOC*-causing glaucomas. For this we compared the 1.5X altered genes from wild-type (Adh.MYOCWT) and Adh.Mutants using the Venn map feature of the GS10 program. Because this feature uses the Affymetrix ID numbers for the comparisons, for this analysis we used 1.5-fold redundant gene lists (all spots for a given Entrez number included). Fold change ≥1.5 lists of each of the wild-type and mutants versus the null virus were created as indicated in methods, exported to the hard drive, and cleaned of IDs that did not have an Entrez number.

**Figure 6:**
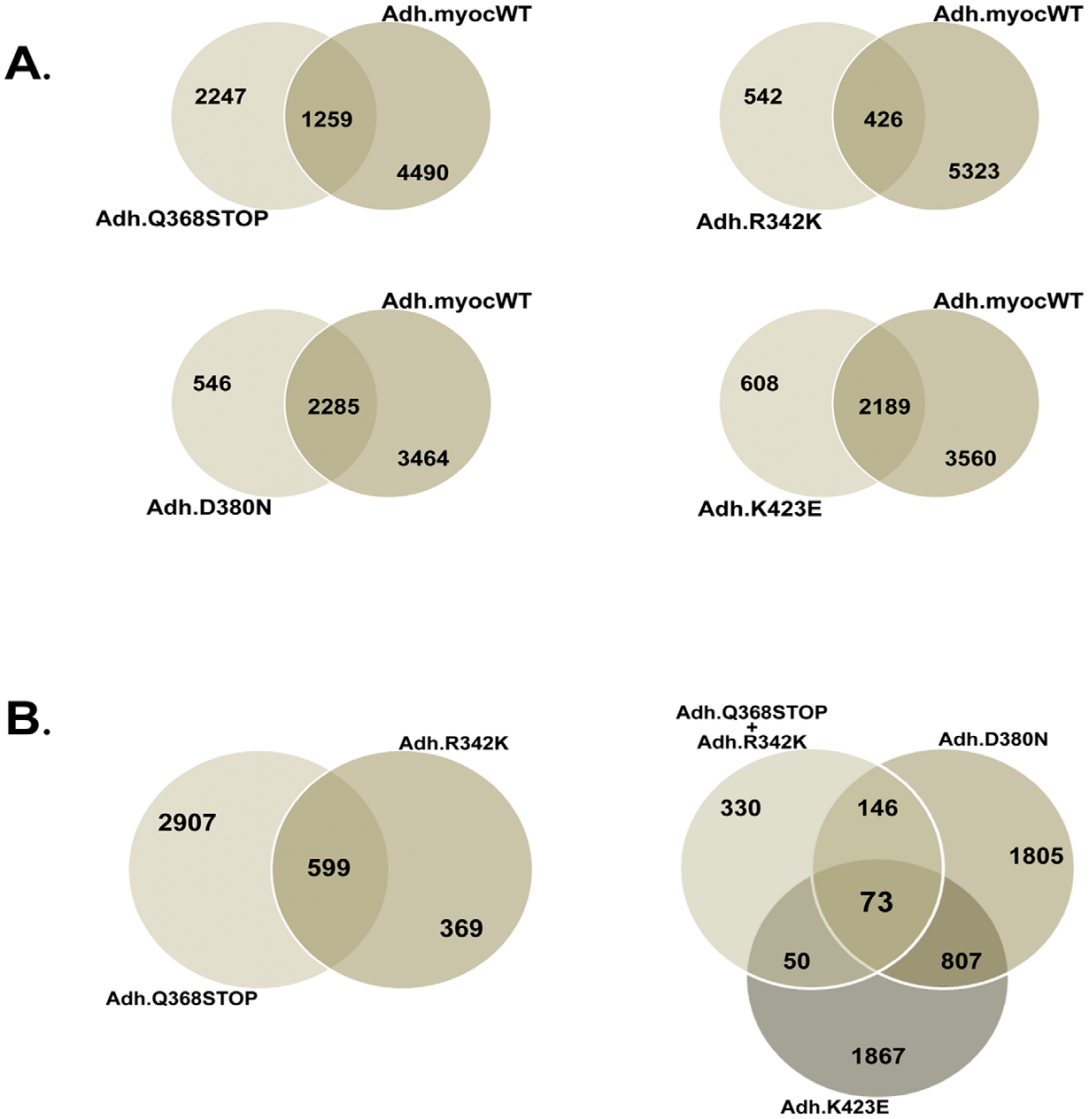
*MYOC* mutants shared different gene percentages with *MYOC* wild-type, and shared 73 genes among themselves. Venn diagrams of genes differentially altered 1.5-fold in response to overexpression of *MYOC* wild-type and *MYOC-*mutants. Each circle represents one condition. Intersections indicate numbers of genes that are shared between the different conditions. A) genes shared of each of the mutants with the wild-type. B) genes shared by all mutants.

To first get an insight on the extent of sharing changes between the wild-type and the mutants, re-imported all Entrez lists were Venn mapped comparing each of the mutants to the wild-type (Figure 6A). This comparison showed that the number of altered genes in each mutant that did not overlap with those altered in the wild-type varied. The stop mutation Q368X contained the highest number of non-overlapping genes, both in absolute numbers and in percentage (2,247; 64%) (Figure 6A). The mutation R342K, whose absolute number of altered genes is the lowest of the mutants studied (Figure 2), contained 542 non overlapping genes, which amounts to a still high percentage of the total (56%) (Figure 6A). The other two mutants (set #2), D380N and K423E had a lower number of non-overlapping genes (24% and 22% respectively), sharing more of the changes with the wild-type.

To study the number of genes which were simultaneously altered in all mutants, we Venn mapped their FC ≥1.5 lists (Figure 6B). We found that a total of 73 genes were altered in all four mutants (Figure 6B).

### Enriched Functions of the Mutant Overlapping Gene List

**Figure 7:**
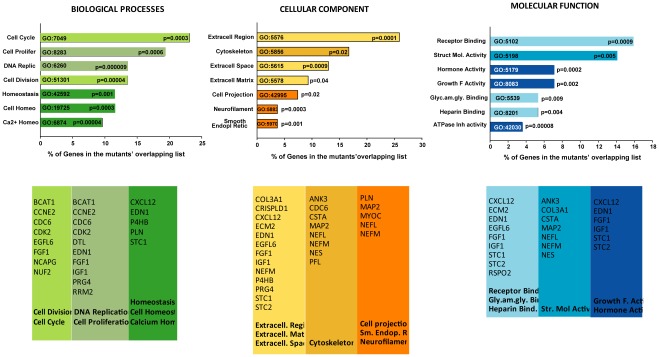
Gene ontology. The ECM category (GO:5576) contained the highest percent and significance of MYOC-induced genes. *Top: e*nriched GO categories of the 73 gene list shared by all mutants (from Figure 6) sorted by *P* values. *Bottom*: genes selected from the categories shown on top (color coded).

To investigate functions potentially involved in a common causative action of the four mutants studied, we searched for the GO enriched functions in the 73 mutant overlapping gene list. We analyzed the three GO SLIMS major functional categories of Biological Processes, Cellular Components and Molecular Functions, and sorted them by percent of genes in each of their subcategories which were statistically significant (P≤0.05). The diagram of the top seven subcategories from each main group is shown in Figure 7 top. In Biological Processes, cell cycle (GO:7049) contained the highest percent of genes number (23.1%, P≤0.0003,) and DNA replication (GO:6260) was the most significant (P≤0.000009, 13.5% genes). In Cellular Components, the extracellular region subcategory (GO:5576) was both the one with highest percentage of genes and most significant (25.9%, P≤0.0001). In the Molecular Function category, the subcategory with more genes was that of receptor binding (GO:5102) (15.8%, P≤0.0009) while the one most significant was the ATPase inhibitor activity (GO:42030) (P≤0.00008, 3.5% genes) (Figure 7 top).

Genes selected from each of the lists of the subcategories are shown in Figure 7 lower panel. As expected, some of these genes coincide with those selected above through other selection means, ratifying the relevance of their potential involvement in the association of *MYOC* mutants with glaucoma. Among the genes, we observed the signaling cytokine *CXCL12*, the vasoconstrictor *EDN1*, the extracellular matrix constituent *ECM2*, the cysteine protease *CSTA*, the calcium homeostasis proteins *STC1*, *STC2* and the growth factors *IGF1* and *FGF1*. It is likely that a different extent combination of the functions encoded by these genes is what it would cause the *MYOC* mutants to trigger the development of the disease.

### Genes Altered in All Mutants and not in the Wild-type. Identification of Cystatin A

**Figure 8:**
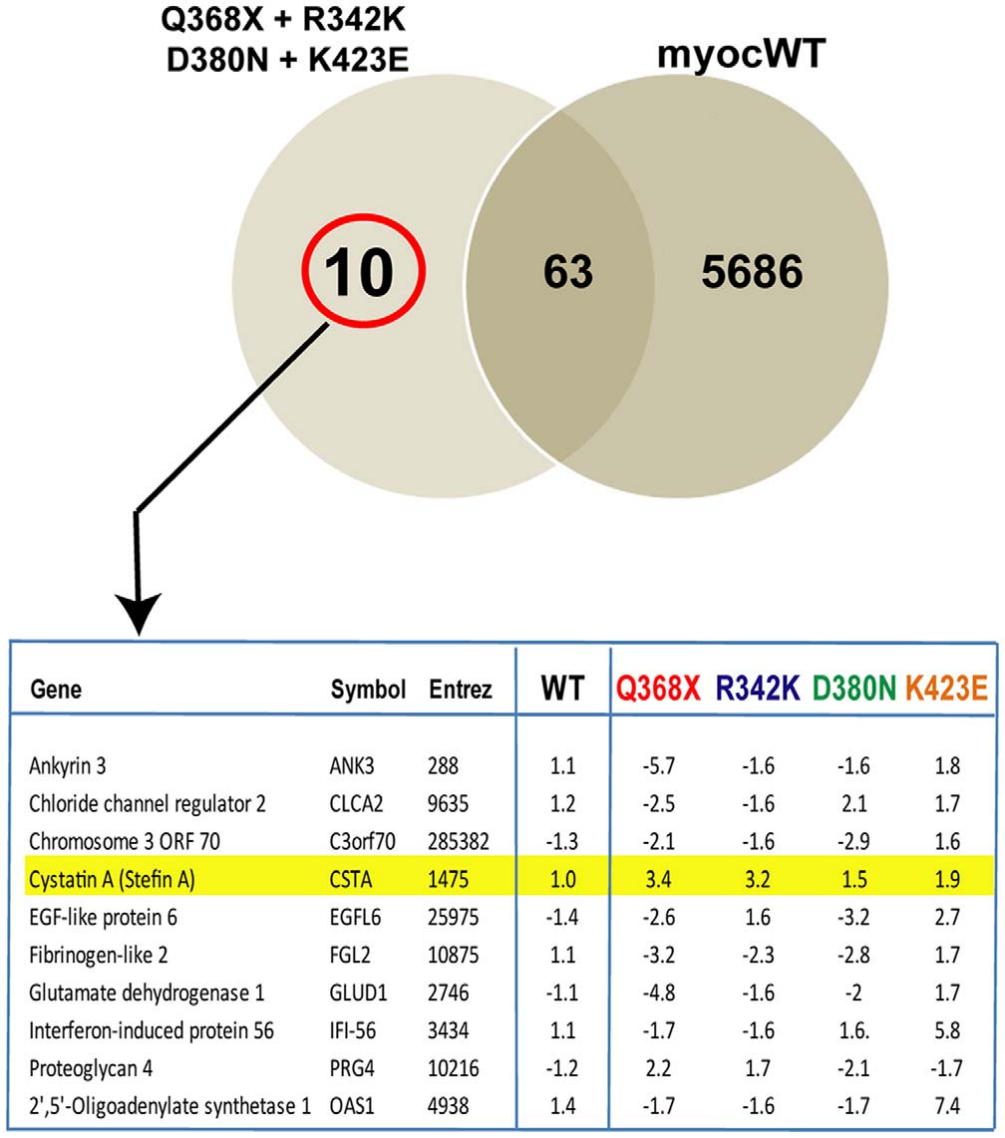
Cystatin A is induced in all *MYOC* mutants and not in *MYOC* wild-type. *Top*: Venn diagram comparing genes altered 1.5-fold in all *MYOC* mutants (left circle) with those altered in the *MYOC* wild-type (right circle) (from Figure 6). Intersection indicates numbers of altered genes which are shared between all the mutants and those altered in the wild-type. Number of genes specifically altered by the mutants is outside the intersection and circled in red. *Bottom*: list of specific genes highlighting Cystatin A and its altered fold change values in each condition.

Next we were interested to investigate whether the genes altered in all mutants were also altered in the wild-type. To determine how many of the 73 genes in the mutants’ overlapping list were mutant-specific, we performed a Venn map comparing the shared mutant list with that of the 5,749 genes altered by the wild-type. Looking for non-overlapping genes, we found that only 10 out of the 73 genes were altered specifically in all mutants and were not shared with the altered genes of the wild-type (Figure 8).

Analysis of the functions of the 10 specific genes revealed the presence of a cysteine protease inhibitor, *CSTA(*alias *Stefin A*), which showed a no change 1.0-fold in the wild-type and an increase of 3.4-, 3.2-, 1.5- and 1.9-fold in Q368X, R342K, D380N and K423E respectively. The induction of CSTA in all cells overexpressing the MYOC mutants was validated by TaqMan PCR in a different HTM primary cell line (HTM-137) and using new viral stocks. Cells were infected with each of the four mutants, Ad5.CMV-Null and Adh.MYOCWT as indicated in methods. RNA was extracted 48 h post-infection and its cDNA assayed for expression of *CSTA*, using *MYOC* as a positive control and 18S as the endogenous calibrator. Results were expressed in FC values of each of the genes in the treated (MYOCs) over negative control (Ad5.CMV.Null), and normalized to their own 18S. In cells infected with Q368X, R342K, D380N and K423E, *CSTA* was induced 4.6-fold (P = 0.012), 3.7-fold (P = .001), 6.45-fold (p = 0.015), and 5.7-fold (P = 0.002) respectively. In cells infected with Adh.MYOCWT, rather than a no change as it occurred in the chip, *CSTA* was reduced −3.0-fold (P = 0.0001), a result most likely due to the highest sensitivity to the TaqMan assay. The overexpression of *MYOC* in each of the dishes (mutants and wild-type infected) was used as a positive control and ranged from 891 to 7,131 FC over that in Ad5.CMV-Null.

### Functional Assay of Cystatin A. Involvement in Wild-type Myocilin Processing

**Figure 9:**
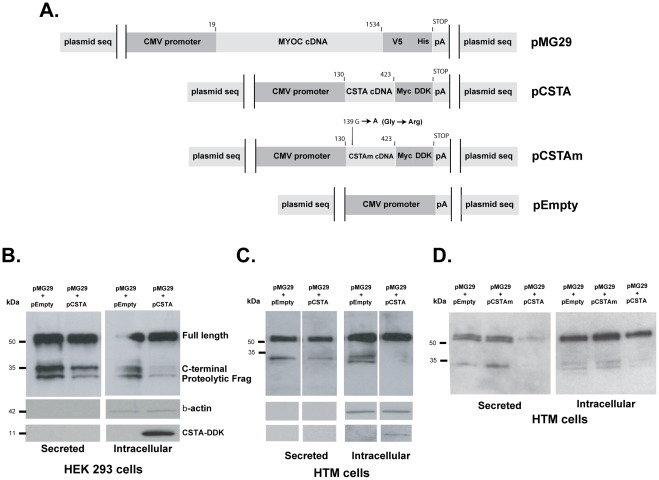
Cystatin A inhibits the processing of MYOC wild-type in cultured cells. Recombinant expression plasmids containing tag-fused full coding wild-type *MYOC* (pMG29), *CSTA,* and controls plasmids, inactive mutated *CSTA (CSTAm)* and pEmpty, were generated as indicated in Methods. pMG29 was co-transfected with either pCSTA, pCSTAm or pEmpty (1∶2) and harvested at 48 h post-transfection. Equivalent volumes of cell extracts and of their supernatants were loaded onto 4–15% SDS-PAGE gels, transferred to PVDF membranes and analyzed by immunoblotting. Different MYOC protein forms (full length and processed) were detected with an anti-V5 mouse monoclonal followed by an anti-mouse horseradish peroxidase antibodies. Blots were re-probed with β-actin and DDK antibodies for loading and identification controls. Percent of the MYOC processed band was calculated by densitometry. A) schematic representation of the expression cassettes of the recombinant plasmids. B, C and D: Representative western blots with extracts from transfected cells. B) extracts from HEK293 co-transfected by calcium phosphate. C and D) extracts from primary HTM-137 cells co-transfected by nucleofector electroporation.

In order to investigate whether overexpression of the selected *CSTA* gene would affect the processing of the wild-type *MYOC* protein we generated tagged recombinant plasmids, containing both genes (Figure 9A). Plasmid pMG29 contains 1513 nt of wild-type *MYOC* fused to the V5 tag in a pCDNA3.1D V5-His-TOPO background. Plasmid pCSTA contains the full coding *CSTA* cDNA (Origene) fused to the DDK and MYC elements. Plasmid pCSTAm derives from pCSTA but contains a mutation at nucleotide 139 (G to A) that converts N-terminal Gly-4 residue to an Arginine and inactivates the binding of the inhibitor to the cathepsin B protease [37]. Plasmid pEmpty does not contain any transgene cDNA (see methods) and was used as a control. pMG29 was co-transfected with either pCSTA or pEmpty in HEK293 and primary HTM-137 cells in a ratio of 1∶2 (Figures. 9B and 9C). Extracted intracellular and secreted proteins were run in PAGE gels, blotted and cross-reacted with an antibody against the V5 tag fused to MYOC. Blots were then re-probed with beta-actin for equal loading control and with anti-DDK antibody to confirm the presence of CSTA. The 35 K processed C-terminal appeared as a doublet. In both cell lines, the processing of the wild-type was reduced in the presence of the CSTA protein.

In the HEK293 cells secreted fraction, densitometry of the wild-type and processed C-terminal fragments showed a mean density of the processed MYOC bands to be a 48.6% of the total secreted MYOC in the cells transfected with pEmpty and 36.4% in the cells transfected with pCSTA (a percent reduction of 25.1%). The difference was more marked in the intracellular fraction where the processed MYOC was 49.8% of the total in the control and 12.5% in the cells transfected with *CSTA* (a percent reduction of 74.9%).

In the primary HTM cells secreted fraction, densitometry of the wild-type and processed C-terminal fragments bands showed the mean density of the processed MYOC to be 33.0% of the total secreted MYOC in the cells transfected with pEmpty controls and 17.2% in the cells transfected with pCSTA (a percent reduction of 47.9%). The difference was more marked in the intracellular fraction where the processed MYOC was 36.5% of the total in the control and 6.6% in the cells transfected with *CSTA* (percent reduction of 89.1%). Protein extracts were run in duplicate gels and transfections were repeated on the HEK293 to confirm the findings. Confirmation of the processing effect of CSTA in wild-type MYOC was achieved by the transfection and overexpression of HTM-137 cells with pCSTAm, a plasmid where the activity of the CSTA protein is destroyed by the mutation of the conserved residue Gly-4 to Arg [37] (Figure 9D). In the secreted fraction, densitometry readings of the mutant experiments showed the processed MYOC in cells transfected with pEmpty and pCSTAm to be 23.6% and 37.1% respectively of the total secreted, while in cells transfected with pCSTA was 10.5% (percent reductions of 55.5 and 71.7% respectively). In the intracellular fraction, the processed MYOC was 17.2% and 20.4% of the total MYOC in cells transfected with pEmpty and pCSTAm and 9.5% in cells transfected with pCSTA (percent reductions of 44.8% and 53.4% respectively).

These results indicate that the increased levels of the protease inhibitor CSTA, but not those of the inactivated CSTA, are able to inhibit the processing of MYOC. In both cell types the inhibition occurred in both fractions and the reduction seems to be higher in the non-transformed trabecular meshwork specific cells than in the transformed embryonic kidney HEK293 line. Therefore the previously reported protein processing reduction observed in the MYOC mutants [28] might be due to overexpression of *CSTA*. The finding could lead to a common treatment of the inhibition of *CSTA* to ameliorate the development of the disease caused by *MYOC* mutants.

## Discussion

Our goal in this study was to gain insight into the molecular mechanisms linking *MYOC* mutants to the development of glaucoma. Even if the *MYOC* gene is expressed in several non-ocular tissues, *MYOC* mutants are associated to only one disease, glaucoma. Because myocilin is induced by a variety of stress factors (from oxidative stress to mechanical strain), it is very likely that the disease caused by the *MYOC* mutants would be aggravated by their induced overexpression under stress conditions. To date, a few studies have addressed the effect of overexpressing some *MYOC* mutants in ocular and non-ocular cells [15], [18], [23], [28]. However, except for one study in fruit flies [19], most of them focused on the effect of the mutants in a few cellular mechanisms. Because *MYOC* mutants cause high tension glaucoma in humans, and because an elevated IOP phenotype is the result of a dysfunctional trabecular meshwork, here we studied the effect of overexpressing them in primary non-transformed, human trabecular meshwork cells. With the intent of uncovering new mechanisms, our approach entailed the examination of global changes induced by the mutants in the entire transcriptome of the cell.

### Genes, known and New

When we examined the most altered genes induced by all four mutants, we observed a high number of genes which had been previously identified as functionally relevant for the trabecular meshwork at either physiological or glaucomatous stress conditions. Among those genes are *EDN1*, *MGP*, *IGF1*, *CALR*, *PDIA4*, *PCLOCE2*, *MMP1*, *MMP3*, *SFRP1*, *FN1*, *PDPN*, *OGN, OSF2,* THBS2, *THBD*, LOX, LOX-L1, *TAC1, Secretogranin II* (*SCG2*) and *SNCA*. Some of these had already been reported to be associated with *MYOC* mutants (e.g. *MMP1*, *CALR*, and *PDI*) or wild-type (SFRP1, *FN1*) in other studies [18], [19], [41] [25], [63], and validates the finding. However, the association of most of them with *MYOC* mutants was unknown.

As is not uncommon in this type of studies, some of the found genes appeared to serve a counteracting purpose while others appeared to be directly related to the detrimental effect. For example, *END1* was on the top downregulated list in all four mutants. Endothelin protein is processed to secrete a potent vasoconstrictor peptide with a well-established connection to glaucoma [64]. Endothelin can lower IOP by contracting the ciliary muscle, but conversely, an antagonist of its receptor can also lower IOP in glaucomatous monkeys [64]. The endogenous *EDN1* downregulation by myocilin mutants seen here is curious. It could be interpreted as either a signal for contributing to the high IOP phenotype of the mutants or to a counteracting effect.

A gene that could enlighten the correlation between the mutants and elevated IOP is *MMP1*. In this study all mutants but one (Q368X) downregulated *MMP1*, while the wild-type upregulated its expression. Matrix metallopeptidase 1 breaks down collagen type 1, an important component of the ECM of the outflow tissue. Increased levels of *MMP1* have been extensively associated with increasing outflow facility and lowering of IOP [41], [65]. Therefore, the *MMP1* downregulation observed here could be seen as a contribution to the build-up of an ECM excess, increase of aqueous humor resistance and consequent elevated IOP produced by the mutants. The unexpected upregulation of *MMP1* by Q368X (confirmed repeatedly by TaqMan PCR) could then be explained as one of the molecular reasons as to why this mutation causes a milder outcome of the disease. Curiously, we had previously reported an interesting feedback loop regulation of expression between the *MMP1*/*MYOC* genes [31], [41], [66], where overexpression of wild-type *MYOC* induced the expression of *MMP1* [31] while overexpression of *MMP1* reduced that of wild-type *MYOC* [41], [66]. It is possible that a homeostatic balance between these two proteins would be disrupted by the presence of the mutated protein form.

Another potentially relevant gene found in these studies was *PDIA4*. In an earlier report overexpressing Q368X in trabecular meshwork cells, Kee and co-workers [18] had observed increased PDI levels in the treated cells. In this study PDIA4 expression was induced by both the Q368X and R342K mutants, but not by D380N and K423E. This enzyme which catalyzes the formation and breakage of disulfide bonds is a key component of the protein folding mechanism. Myocilin folding and complex formation is relevant for its function and has been known to be mediated by the formation of covalent disulfide bonds involving its five cysteine residues [67]. Although the fifth residue 433Cys is not present in the Q368X stop mutation, the induction of the PDI is bound to affect the stability of the proteins and even perhaps affect the secretion of other proteins [68]. In our early work, *PDIA4* was induced in response to elevated pressure [45], [46]. It is known that mechanical strain causes deformation of proteins, triggering them to unfold and subsequently refold [69]. Protein disulfide isomerase induction could thus be a mediator of this effect and represent a cellular defense response against the altered protein, as it also occurs in Creutzfeld-Jacob disease [70]. Why *PDIA4* it is only induced by two of the mutants is not yet clear and might be a sign of the different avenues through which each mutant contributes to the disease.

Some of the genes altered by these mutants were new. These include among others RAB39B, *STC1*, *CDH15*, *CXCL12*, *CSTA*, *ECM2* and *Lysosomal-associated membrane protein 3* (*LAMP3*), a gene which is expressed in lymphoid organs and dendritic cells. Some of these genes were commonly altered in all mutants while others where changed only in one or two of them. Their encoded functions could play a role on the mutant’s linkage to the disease. Thus, *RAB39B*, upregulated in all mutants, is a small GTPase binding protein described as being specific to neurons and involved in vesicular trafficking [71]. This mechanism, which includes the ability to secrete the protein, has been shown to play a key role in myocilin function [12], [13] and was suggested to be disrupted by *MYOC* mutants [11]. It would be possible that upregulation of RAB39B could have a role on the trafficking and non-secretion of the MYOC mutants.

Another new gene, *STC1,* was in contrast downregulated in all mutants. Stanniocalcin regulates calcium and phosphate transport in the kidney and has been postulated to prevent hypercalcemia [72]. The downregulation of *STC1* could be an indication that the *MYOC* mutants are favoring a trend towards calcification of the trabecular meshwork.

Few of the genes encoding proteins reported to interact with Myocilin made the 1.5X FC cutoff. Two of them did. Secreted frizzled protein 1, [25] which was downregulated in mutants Q368X and R342K, and *FN1* [63], that was upregulated in D380N and K423E. The *SNCA* gene which was also upregulated in D380N and K423E is from the same family as the *MYOC* binding protein *γ*-synuclein, but in contrast, showed no co-localization and thus, not binding [73]. α-Synuclein, is a protein abundant in neurons and involved in Parkinson and other neurodegenerative disorders and in here could play the role of a pathological chaperone, as it has been described in other systems [74]. α-Synuclein peptides are a major component of amyloid plaques in the brains of patients with Alzheimer’s disease [75], which has been speculated to have some common mechanistic features with Glaucoma [76].

### Differences between Mutants

Overall, there were many similarities between Q368X and R342K (which we termed set #1). Those similarities were distinct from the changes induced by the other two mutants D380N and K423E (set #2). This was an unexpected finding, since there are not described phenotypic similarities shared between the mutants of the same group, or described phenotypic differences between the two groups. At the moment we can postulate that, in addition to IOP, there may yet be undefined glaucoma biomarkers in the altered genes pool which could differently influence the development of the disease in sets #1 and #2.

A look at the genes representing trabecular meshwork relevant functions and physiological markers of glaucoma by the use of heat maps (Figures 4 and 5) revealed that most of genes in those lists are affected by overexpression of *MYOC* mutants. The trend of similarities between the two sets of mutants is clearly seen in the genes involved in calcification, WNT signaling and stress, while it is less obvious in those genes encoding collagen-elastin cross-linking and in the biomarkers’ list. The intensity of the changes was considerably greater in the stress and UPR function group, especially for the Q368X mutation. This mutation clearly downregulated genes that could induce elevated IOP, such *TGM2* and *SFRP1* [26], [60] while inducing others that have a role in protein folding, such as *CALR* and *Calnexin* (*CANX*). This combined regulation could be beneficial and contribute to the milder outcome of the disease in the Q368X mutant. In contrast, the lower induction of the same genes (*CALR* and *CANX*) by R342K, together with the upregulation of the calcification genes *OGN* and *OMD* (heat map Figure 4) could provide this mutant (otherwise similar to the stop mutation) with the difference needed to induce an elevated pressure.

Although each of the *MYOC* mutants exhibited individual characteristics which are most likely instrumental in triggering different disease severities, there was a need to search for a common thread. In a Venn map comparison with wild-type we observed that the four *MYOC* mutants exhibited a different percentage of non-shared altered genes. Set #1 mutants had a larger non-shared percentage than set #2. This could be an indication that set #1 would be further away from the proposed protective mechanism of the wild-type than set #2 or, that these mutants require a higher number of changes to cause the disease. When comparing the four mutants against themselves, we found only 73 altered common genes. And from the 73, only 10 were not altered in the wild-type.

### Cystatin A as a Potential Common Link

One of the genes found elevated in all mutants and not elevated in the wild-type was *CSTA,* which encodes for the cysteine protease inhibitor cystatin A. This gene is present in most tissues, its expression is associated with tumor growth and it is a serum biomarker for screening cancer patients. The upregulation of the *CSTA* mRNA by the *MYOC* mutants is intriguing. Because the protein involved in the processing of myocilin, calpain II, is a cysteine protease, and because myocilin cleavage is inhibited by MYOC mutants, one could reasonably infer that *CSTA* plays a role in the inhibition of the process. Whether the elevation of cystatin A seen here can result also in the inhibition of calpain is not yet known. Cystatin A is known to inhibit papain and cathepsins, not calpains. However, a recent study investigating induced cell death in monocytes and macrophages has shown that calpain activation occurred downstream of cathepsin B (a CSTA substrate) [77]. They concluded that cathepsin B activated calpain [77]. Such finding would implicate that increased levels of cystatin A would, by inactivating cathepsin B, result in the inactivation of calpain. In our functional assay, overexpression of cystatin A reduced the processing of wild-type myocilin while overexpression of an inactive CSTA did not. This effect could then have been achieved by either a downstream inactivation of calpain through the inactivation of cathepsin B, or by a direct cleavage of myocilin by cathepsin B.

It is important to point out that *MYOC*-causative glaucoma occurs only in heterozygous individuals, and that *MYOC* forms wild-type/mutant hetero-oligomers which lead to the formation of insoluble aggregates (gain of function) [12], [17]. It would be logical to assume that myocilin processing, affected by the expression of the mutants, plays a fundamental role in the formation of the hetero-aggregates. The fact that hetero-aggregation could also modulate the extracellular environment [15] provides an additional support for the relevance of faulty hetero-aggregates in the development of glaucoma.

Finally, although several mechanisms are bound to be involved in the association of *MYOC* mutants to glaucoma, it looks that activation of cysteine protease inhibitors could be a common, general one. It would be interesting to investigate whether the application of an inhibitor to *CSTA*, such as its siRNA, could restore the normal *MYOC* processing and affect the outcome of the disease. Our results showing that overexpression of an inactive CSTA reverted the decrease processing would support this possibility. It would also be of interest to determine whether a screening of CSTA levels in the serum could be applied to glaucoma as it is presently occurring in cancer [39].

### Conclusions

Our study on the transcripts altered by overexpression of *MYOC* mutants in glaucoma-relevant primary human cells provides key insights on the potential mechanisms leading to the development of *MYOC*-linked glaucoma. We uncovered that each mutant’s phenotype could result from its unique effect on the transcriptome. We learned that a number of important genes which have been historically associated with physiological and pathological mechanisms of the human trabecular meshwork are altered by the expression of these MYOC mutants. On overall mechanisms, we identified that, genes of the UPR pathway were the most affected. On individual gene analyses, we confirmed the involvement of previously MYOC-associated genes (e.g. *MMP1, PDIA4, CALR, SFPR1*) and revealed the relevance of some new ones (e.g. *STC1, RAB39B*, *CXCL12*). Most importantly, we discovered a mutant-specific induced gene, *CSTA.* This inhibitor of cysteine proteases was functional, and inhibited MYOC protein processing in cultured cells. We believe these findings do significantly impact our understanding of *MYOC*-caused glaucoma and could provide the basis for the potential development of a broad-spectrum therapy for the mutant disease.

## Supporting Information

Table S1 **Q368STOP. The 100 most altered genes.** a) 50 upregulated; b) 50 downregulated
(XLSX)

Table S2 **R342K. The 100 most altered genes.** a) 50 upregulated; b) 50 downregulated
(XLSX)

Table S3 **D380N. The 100 most altered genes.** a) 50 upregulated; b) 50 downregulated
(XLSX)

Table S4 **K423E. The 100 most altered genes** a) 50 upregulated; b) 50 downregulated
(XLSX)

Table S5 **Taqman FC and p-values of selected relevant genes.** Comparison to microarray FC
(XLSX)

Table S6 **Physiological Biomarkers List for Glaucoma (Trabecular Meshwork).**
(XLSX)
